# Transcriptional and metabolic rewiring of colorectal cancer cells expressing the oncogenic KRAS^*G13D*^ mutation

**DOI:** 10.1038/s41416-019-0477-7

**Published:** 2019-05-28

**Authors:** Theodosia Charitou, Sriganesh Srihari, Miriam A. Lynn, Mohamed-Ali Jarboui, Erik Fasterius, Max Moldovan, Senji Shirasawa, Toshiyuki Tsunoda, Marius Ueffing, Jianling Xie, Jin Xin, Xuemin Wang, Christopher G. Proud, Karsten Boldt, Cristina Al-Khalili Szigyarto, Walter Kolch, David J. Lynn

**Affiliations:** 1 EMBL Australia Group, South Australian Health and Medical Research Institute, North Terrace, Adelaide, SA 5000 Australia; 2 Institute for Ophthalmic Research, University of Tübingen, Tübingen, Germany; 3 Werner Siemens Imaging Center, University of Tübingen, Tübingen, Germany; 4 School of Biotechnology, Royal Institute of Technology, Stockholm, Sweden; 5 Faculty of Medicine, Fukuoka University, Fukuoka, Fukuoka Prefecture 814-0133 Japan; 6 Nutrition, Diabetes & Metabolism, South Australian Health & Medical Research Institute, Adelaide, SA 5000 Australia; 7 School of Biological Sciences, University of Adelaide, Adelaide, SA 5000 Australia; 8 Systems Biology Ireland, University College Dublin, Dublin, Ireland; 9 School of Medicine, University College Dublin, Dublin, Ireland; 10 Conway Institute, University College Dublin, Dublin, Ireland; 11 School of Medicine, College of Medicine and Public Health, Flinders University, Bedford Park, SA 5042 Australia

**Keywords:** Cancer genomics, Gene regulatory networks

## Abstract

**Background:**

Activating mutations in KRAS frequently occur in colorectal cancer (CRC) patients, leading to resistance to EGFR-targeted therapies.

**Methods:**

To better understand the cellular reprogramming which occurs in mutant KRAS cells, we have undertaken a systems-level analysis of four CRC cell lines which express either wild type (wt) KRAS or the oncogenic KRAS^G13D^ allele (mtKRAS).

**Results:**

RNAseq revealed that genes involved in ribosome biogenesis, mRNA translation and metabolism were significantly upregulated in mtKRAS cells. Consistent with the transcriptional data, protein synthesis and cell proliferation were significantly higher in the mtKRAS cells. Targeted metabolomics analysis also confirmed the metabolic reprogramming in mtKRAS cells. Interestingly, mtKRAS cells were highly transcriptionally responsive to EGFR activation by TGFα stimulation, which was associated with an unexpected downregulation of genes involved in a range of anabolic processes. While TGFα treatment strongly activated protein synthesis in wtKRAS cells, protein synthesis was not activated above basal levels in the TGFα-treated mtKRAS cells. This was likely due to the defective activation of the mTORC1 and other pathways by TGFα in mtKRAS cells, which was associated with impaired activation of PKB signalling and a transient induction of AMPK signalling.

**Conclusions:**

We have found that mtKRAS cells are substantially rewired at the transcriptional, translational and metabolic levels and that this rewiring may reveal new vulnerabilities in oncogenic KRAS CRC cells that could be exploited in future.

## Background

Colorectal cancer (CRC) is the world’s fourth most-deadly cancer, killing ~700,000 people every year.^1^ A major factor in poor survival outcomes in late-stage disease is the development of drug resistance. It is now well-established that treatment of metastatic CRC with antibodies (Cetuximab and Panitumumab) against the epidermal growth factor receptor (EGFR) is effective only among patients with wild-type KRAS (wtKRAS) carcinomas, whereas mutant KRAS (mtKRAS) carcinomas are resistant to this treatment.^2^

KRAS is a proto-oncogene, encoding a small (21 kDa) guanosine triphosphate (GTP)/guanosine diphosphate (GDP)-binding protein downstream of the EGFR that is involved in the regulation of cellular responses to many extracellular stimuli, in particular mitogens and growth factors such as epidermal growth factor (EGF) and transforming growth factor alpha (TGFα). EGFR ligands, such as TGFα, are often overexpressed in CRC and are thought to contribute to autocrine stimulation of tumour growth and invasiveness.^3^ Mutations in KRAS, which abrogate its GTPase activity and thus result in the constitutive activation EGFR signalling, are found in up to 45% of CRCs.^4^ More than 90% of the mutations in KRAS in CRC patients occur at just three positions: codons 12, 13 and 61. The most common mutations are G12D, G12A, G12R, G12C, G12S, G12V and G13D.^5^ Unfortunately, after more than three decades, KRAS is still considered an undruggable target in the clinic,^4^ and the search is now focused on targeting alternative pathways that are activated in mtKRAS cells, to circumvent or prevent drug resistance.

A key output of activated EGFR signalling is the induction of a complex and highly dynamic set of transcriptional networks which regulate cellular responses such as proliferation, apoptosis, differentiation and migration.^6^ A better understanding of how these transcriptional networks are differentially regulated (rewired) in mtKRAS cells in comparison to wtKRAS cells would provide useful new insight to aid in identifying new therapeutic options to target in mtKRAS cells. Here, we have used RNA sequencing (RNAseq) to profile the transcriptional response, before and after activation of EGFR signalling by TGFα stimulation, of two isogenic HKe3 CRC cell lines engineered to express either KRAS^wt^ or KRAS^G13D^.^7^ RNAseq was also performed on the parental HKe3 cell line and on HCT116 cells, which express KRAS^G13D^ endogenously.^8^ These analyses revealed substantial differences in the transcriptional regulation of metabolic pathways in wtKRAS and mtKRAS cells, which were subsequently validated by metabolomics analysis. Furthermore, mtKRAS cells were unexpectedly highly transcriptionally responsive to activation of the EGFR by TGFα stimulation. In contrast to wtKRAS cells, TGFα stimulation of mtKRAS cells did not lead to a further activation of protein synthesis beyond the elevated basal levels.

## Methods

### Cell culture

The HCT116, HKe3-mtKRAS, HKe3-wtKRAS and HKe3 (parental) cell lines were kindly provided by Dr. Senji Shirasawa, Faculty of Medicine, Fukuoka University, Japan.^7^^,^^8^ DNA was extracted with the DNeasy Blood and Tissue Kit (Qiagen) at 80% confluency (according to the manufacturer’s instructions). The extracted DNA was stored in −80 °C before sequencing. For the RNAseq time course, cells were maintained in DMEM (Dulbecco’s modified Eagle’s medium; Gibco, Australia) supplemented with 10% (v/v) foetal calf serum (Assay matrix, Australia), 2 mM l-glutamine (Gibco) and incubated in a humidified atmosphere of 5% CO_2_ at 37 °C. Cells were serum starved for 18 h and subsequently stimulated with TGF-α (0.01 μg/mL; Abcam cat no. ab9587) for 15, 30, 60, 90 and 120 min. Three biological replicates were assessed at each time point.

### Whole-genome sequencing

Cell line authentication was initially performed by RNA-seq as previously described,^9^ and each cell line also underwent whole-genome sequencing. Library preparation for whole-genome sequencing was done with the TruSeq DNA PCR-free Kit (Illumina), followed by sequencing on a HiSeqX instrument. Read alignment to the GRCh38 assembly was done with the BWA (v0.7.13) software,^10^ followed by de-duplication, base quality score recalibration and clean-up using the Genome Analysis Toolkit (GATK, v3.5.0).^11^ Single-nucleotide variants were called using the HaplotypeCaller GATK module, and subsequently annotated with snpEff (v4.2).^12^ The resulting variants were filtered with GATK’s standard parameters, in addition to filtering for a total depth of at least 10.

### Active KRAS pulldown assay

Active KRAS pulldown was performed using a K-Ras activation assay kit (Cell Biolabs) as described in the product manual; HA-tagged KRAS was provided with the kit.

### Cell proliferation assay

Fifty thousand cells/well were seeded in six-well plates and grown in DMEM containing 10% foetal bovine serum (FBS) for 72 h. Cells were washed twice with phosphate-buffered saline (PBS) and stained with 1 mL 0.2% crystal violet (Sigma) in 20% methanol for 10 min. Excess staining solution was removed by three H_2_O washes, and plates were dried completely. The crystal violet retained by the cells was solubilized with 0.5 mL 10% acetic acid solution by gentle shaking for 20 min. Solubilized crystal violet was diluted 1:5 in H_2_O and absorbance was measured at 590 nm against 2% acetic acid solution as blank. Experiments were performed in triplicate.

### Colony-forming assay

Five thousand cells were seeded in 100 mm dishes in DMEM supplemented with 10% FBS. Media was replenished every 72 h. When colonies became microscopically visible, cells were washed twice with PBS and stained with 0.2% crystal violet (Sigma) in 20% methanol for 10 min. Cells were washed three times with H_2_O to remove excess stain and dried completely before being imaged. ImageJ was used to quantify colonies. All experiments were carried out in triplicate.

### Scratch-wound healing assay

The scratch-wound healing assay was carried out as described in ref. ^13^ Briefly, 10,000 cells were seeded in 12-well plates in DMEM supplemented with 10% FBS and allowed to incubate until they reach monolayer confluency. Media was removed, and the cell monolayer was wounded by scratching the culture well surface using a 100 μL pipette tip. The scratch-wounded cells were washed with 1× PBS to remove any cell fragments or detached cells before incubating in fresh DMEM 10% FBS medium for 24 h. Cells were imaged following the scratch procedure and at 24 h. Images were analysed using ImageJ by measuring the average distance between the wound fronts at three different positions along the wound. Experiments were performed in triplicate.

### Cell proliferation assay following treatment with an AMPK inhibitor

The HKe3-mtKRAS and HKe3-wtKRAS cell lines were plated at 1 × 10^5^ cells in 48-well plates. Cells were serum starved for 18 h and then treated with 10 μM of AMPK inhibitor SBI-020965 (Assay Matrix, cat no. A8715). Thirty minutes later, cells were treated with 0.01 μg/mL TGFα (Abcam cat no. ab9587). Cells were collected at 0, 24 and 48 h following TGFα stimulation and stained with FITC-conjugated Annexin V (Life Technologies) in accordance with the manufacturer’s protocols. Stained cells were added to preloaded tubes with a known density of fluorescent Trucount™ beads (BD Biosciences) and DAPI (BD Biosciences). Data were acquired with a BD LSR Fortessa™ flow cytometer and analysed by FlowJo™. All experiments were carried out in triplicate.

### RNA extraction for time-course RNAseq

RNA was isolated from the cell lines using TRIzol (Thermo fisher, Australia) according to the manufacturer’s instructions. Briefly, cells were homogenised in 500 μL of TRIzol. About 0.1 mL chloroform (Sigma, Australia) was added per 500 μL of TRIzol reagent and mixed vigorously for 15 s. The suspension was spun at 12,000 × *g* for 15 min to separate the aqueous and organic layers. The upper layer (containing the RNA) was collected and an isopropanol precipitation reaction was performed. Briefly, 5 μg of glycogen (Life technologies) and 0.25 mL of 100% isopropanol (Sigma, Australia) were added to the upper layer and incubated for 10 min. A pellet formed when the suspension was spun at 15,000 × *g* for 30 min. The pellet was washed twice in 75% ethanol and resuspended in 50 μL of RNase-free water. All RNA samples were treated with DNase (cat. no. AM1906, Ambion) to remove any contaminating DNA from the purified RNA. Briefly, 2 Units/μL of rDNase I enzyme was added to RNA in 10× DNase I Buffer and incubated at 37 °C for 30 min. The reaction was inactivated by addition of DNase Inactivation Reagent and purified DNA-free RNA was ethanol precipitated from the resultant supernatant.

### RNA integrity and quantification

RNA concentration was determined by spectrophotometry on a NanoDrop 2000 spectrophotometer (Thermo Fisher Scientific). RNA concentrations were assessed in ng/μL. A Bioanalyzer (Agilent 2100) was used to measure the RNA integrity. The Qubit (Thermo Fisher Scientific, Australia) quantification method was used to measure final RNA concentrations before library preparation. Briefly, Qubit Working Solution was prepared by diluting Qubit RNA Reagent 1:200 in Qubit RNA Buffer. Concentrated RNA was diluted to within range of the Qubit assay and 2 μL of sample was added to the working solution and the readout was measured in ng/μL.

### FOS qRT-PCR

FOS-specific primers were designed using NCBI Primer BLAST software and the Roche ProbeFinder Assay Design Software. Five micrograms of total RNA from each sample was reverse transcribed into cDNA using the SuperScriptTM II RT first-strand synthesis Kit (cat no. 18062-022; Invitrogen, Australia). Quantitative real-time PCR (qRT-PCR) was carried out using SYBR Green I (Life Technologies, Australia) as a fluorescent dye, according to the manufacturer’s guidelines. Briefly, each reaction was carried out in a final volume of 35 μL containing 5 ng cDNA, 5 μM forward and 5 μM reverse primer with 2× of Fast SYBR Green Master Mix (Life Technologies, 4309155). The PCR conditions were 95 °C for 1 min, 55 °C for 30 s and 72 °C for 30 s. The CFX Connect Real-Time PCR Detection System was used in this assay. All experiments were carried out in technical triplicate, and results were normalised to two referenced genes: beta-2 micro-globulin and beta-actin RNA levels. Analysis of the qRT-PCR data was carried out using the 2^−ΔΔCq^ method.^14^

### RNA sequencing

Total RNA was converted to strand-specific Illumina-compatible sequencing libraries using the NEXTflex Rapid Directional mRNA-Seq library Kit from BIOO Scientific (Austin, Texas) as per the manufacturer’s instructions (v14.10), by staff at the SAHMRI David R. Gunn Genomics facility. Briefly, 200 ng of total RNA was polyA selected and the mRNA chemically fragmented prior to reverse transcription and second strand cDNA synthesis using dUTP. The resultant cDNA was poly adenylated before the ligation of Illumina-compatible barcoded sequencing adapters. The cDNA libraries were treated with UDG (uracil DNA glycosylase) to degrade the second strand and PCR amplified for 15 cycles prior to assessment using a TapeStation 2200 (Agilent) for quality and Qubit fluorescence assay for quantification. In total, 72 cDNA libraries (4 cell lines × 6 time points × 3 replicates per time point) were generated for sequencing. The sequencing pool was generated by mixing equimolar amounts of all sample libraries based on the Qubit measurements. Libraries were sequenced on the Illumina HiSeq 2500 machine (two flow cells) at the SAHMRI David R. Gunn Genomics facility using a v2 High Output 100 cycle Kit (1 × 100 bp SR).

### RNAseq data analysis

The quality and number of reads for each sample were assessed with FastQC v0.11.3 (http://www.bioinformatics.babraham.ac.uk/projects/fastqc/). Adaptors were trimmed from reads, and low-quality bases, with Phred scores <28, were trimmed from ends of reads, using Trimgalore v0.4.0 (http://www.bioinformatics.babraham.ac.uk/projects/trim_galore/). Trimmed reads of <20 nucleotides were discarded. Reads passing all quality control steps were aligned to the hg38 assembly of the human genome using TopHat v2.1.0,^15^ allowing for up to two mismatches. Reads not uniquely aligned to the genome were discarded. HTSeq-count v0.6.0 (ref. ^16^) was used with the union model to assign uniquely aligned reads to Ensembl Hg38.86-annotated genes. Data were normalised across libraries by the trimmed mean of *M*-values (TMM) normalisation method, implemented in the R v3.2.1., Bioconductor package, EdgeR v3.10.2.^17^ Only genes that had at least five reads per million in at least one cell line were analysed for evidence of differential gene expression. Differentially expressed genes were identified using the glm model implemented in EdgeR and were defined as having a log_2_ fold change in gene expression >1 and a Benjamini and Hochberg corrected *P* values of <0.05.^18^ Pathway and Gene Ontology analyses were performed in InnateDB.^19^ A transcription factor-binding site (TFBS) analysis was undertaken using the findMotifs.pl program in HOMER v4.8,^20^ with the human hg38 promoter set to identify enriched motifs. The RNASeq data are available in the NCBI GEO database under accession GSE105094 and GSE110649.

### Targeted metabolomics

For targeted metabolomics analysis of cell lines, the absoluteIDQ p180 kit (Biocrates Life Sciences, Innsbruck, Austria) was used. The p180 kit allows for the simultaneous quantification of 188 endogenous metabolites from six compound classes [21 amino acids (AA), 21 biogenic amines (BA), 40 acylcarnitines (AC), 90 glycerophospholipids (phosphatidylcholines, PC), 15 sphingolipids (sphingomyelins, SM), and the total amount of hexoses (H1) (carbohydrates metabolism)]. The sample preparation and measurement were performed according to the manufacturer’s recommendation. Briefly, cells were cultured in six-well plates in six replicates in standard DMEM + 10% FBS medium until 70% confluency and then serum starved for 8 h. Cells were washed twice with ice-cold PBS, scraped off the plate and collected by centrifugation at 500 × *g* for 5 min at 4 °C. A total of 5 × 10^6^ cells were used for metabolite extraction using 300 μl liquid chromatography mass spectrometry (LC-MS) grade ice-cold MeOH (Millipore). Cells were resuspended in 80% MeOH, vortexed for 20 s twice, and centrifuged at 1500 × *g* for 5 min at 4 °C. Supernatants from six biological replicates for each cell line were collected and stored at −80 °C for further use. Ten microliters of sample was used for metabolomics analysis. Lipids, acylcarnitines and the hexoses were determined by flow injection analysis-tandem mass spectrometry (FIA-MS/MS), while the amino acids and biogenic amines were measured by LC-MS/MS using the AB-SCIEX QTRAP 6500 (Sciex). Data evaluation for the quantification of metabolites and quality assessment was performed with the MetIDQ software (Biocrates Life Sciences) using internal standards and quality control references already spiked into the 96-well kit plate. Quantified metabolites were normalised to the quality controls and metabolites concentration were reported in μM. An ANOVA test was used to identify differentially abundant metabolites between the cell lines.

### Construction and analysis of the weighted gene co-expression network

The WGCNA package^21^ was used to generate a weighted co-expression network using RNAseq gene expression data from the HKe3-wtKRAS and HKe3-mtKRAS cells (36 RNAseq samples). WGCNA computes Pearson correlations between gene pairs using the input gene expression (RNAseq) data and fits this correlation network to the closest scale-free model by reweighting (powering) the similarity measures obtained from correlation values by a parameter *β* (recommended *β* = 7). A weight cut-off (corresponding to a Pearson correlation = ~0.8) was used to build the final network. Hierarchical clustering at a dendrogram cut-off of 0.10 was used to identify modules in the network. The modules were annotated by the top-ranking canonical KEGG pathways and Gene Ontology terms using an overrepresentation analysis. Interactions between modules were summarised by determining the average weight of interactions occurring between genes from different modules, as follows. For any two modules *M*_*i*_ and *M*_*j*_ the weight of the interaction between the modules was computed as: *w*(*M*_*i*_, *M*_*j*_*)* = (sum of weights of interactions between genes in *M*_*i*_ and *M*_*j*_)/(all possible number of interactions between *M*_*i*_ and *M*_*j*_, i.e., |*M*_*i*_| × |*M*_*j*_|), which gave a weight in the range [0, 1]. This resulted in a weighted *module–module interaction network* that summarised the relationships between the modules. Only interactions with weight *w*(*M*_*i*_, *M*_*j*_*)* ≥ 0.5 were retained.

### Protein synthesis measurements

HKe3-wtKRAS and mtKRAS cells were serum starved overnight, preincubated in methionine- and cysteine-free DMEM (Gibco) for 30 min, before stimulation with TGFα (0.01 μg/mL; Abcam cat no. ab9587). One hour later, 10 μCi EasyTag™ l-[^35^S]-methionine (Perkin Elmer, Australia) was added and cells were incubated for further 2 h. Cells were lysed in ice-cold triton lysis buffer [1% (v/v) Triton X-100, 20 mM Tris-HCl pH 7.5, 150 mM NaCl, 1 mM EDTA, 1 mM EGTA, 2.5 mM Na_2_H_2_P_2_O_7_, 1 mM β-glycerophosphate, 1 mM Na_3_VO_4_, 1 mM dithiothreitol and protease inhibitor cocktail]. Incorporated radioactivity was determined by scintillation counting as previously described in ref. ^22^

### Western blotting

Following overnight starvation HKe3-wtKRAS and mtKRAS cells were stimulated with 0.01 μg/mL TGFα (Abcam ab9587) over a time course of 0, 30, 60, 90, 120 and 360 min. There was a minimum of three biological replicates at each time point. Cells were lysed in in 1% NP40 lysis buffer (50 mM Tris-HCl pH 7.5, 150 mM NaCl, 1 mM EDTA, 50 mM β-glycerolphosphate, 0.1%sodium deoxylcholate, 0.1% SDS, supplemented with protease and phosphatase inhibitors). Lysates were cleared by centrifugation at 10,000 × *g* for 10 min and adjusted to equal amounts of protein after measuring protein concentrations using the Pierce BCA assay kit. Equal amounts of lysates were separated by sodium-dodecyl sulfate-polyacrylamide gel electrophoresis (SDS-PAGE) and transferred to nitrocellulose membranes. Blots were incubated with the respective antibodies and developed using a LI-COR Odyssey (Millennium Science) and quantified with Odyssey image studio software.

### Analysis of signalling pathways

Following overnight starvation HKe3-wtKRAS and mtKRAS cells were stimulated with 0.01 μg/mL TGFα (Abcam ab9587) over a time course of 0, 30, 60, 90, 120 and 360 min. There was a minimum of three biological replicates at each time point. Cells were lysed in RIPA buffer. Most antibodies were from Cell Signaling Technology: eIF4E (9742), p-rpS6 (240/244, 2215), p-Erk (4370), Erk (9102), p-PKB (S473, 9271), PKB (4685), p-4E-BP1 (9451), 4E-BP1 (9644), p-ACC (3661), ACC (3662), p-rpS6K (T389, 9209), rpS6K (2708). Antibodies for KRAS (sc-30, F234) and rpS6 (SC74459) were from Santa Cruz Biotechnology; KRAS^G13D^ (26038) was from NewEast Biosciences; and p-eIF4E (PA44528G), anti-β-actin (A2228), MEK1/2 (MAP2K1/2) (M5795) and phospho-MEK1/2 (MAP2K1/2) (M7683) from Sigma-Aldrich.

## Results

**Fig. 1:**
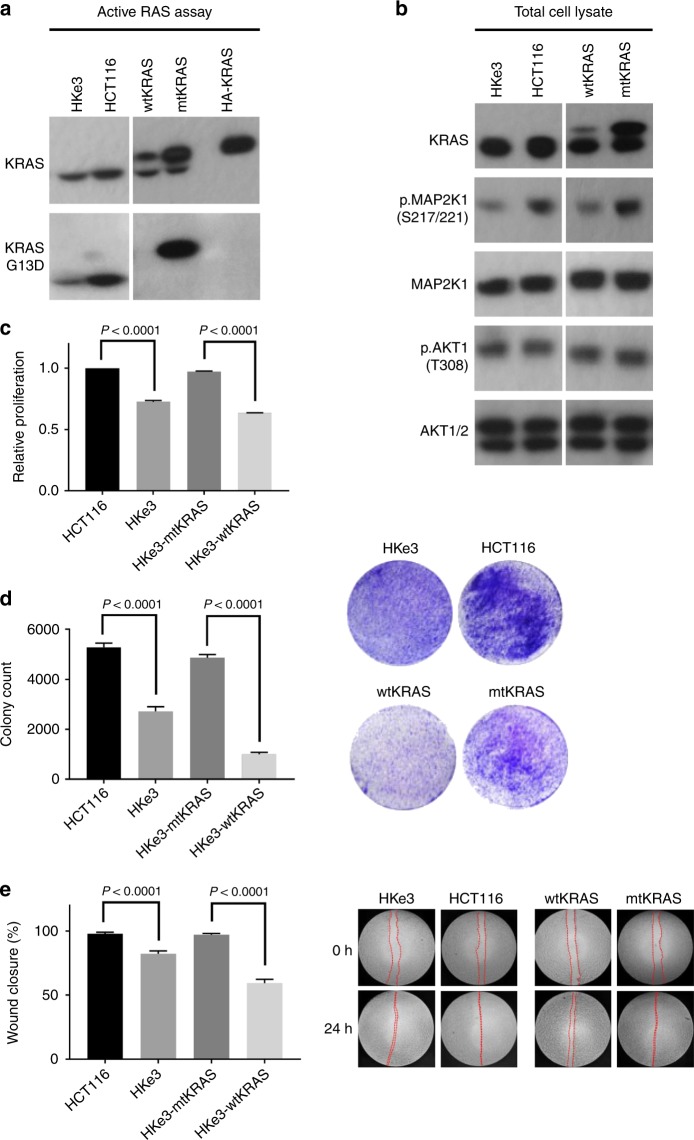
Characterisation of the HKe3-wtKRAS, HKe3-mtKRAS, HKe3 and HCT116 cell lines. **a** The upper band shows a KRAS activity assay, which detects binding of KRAS to the RAS-binding domain of RAF1 (RBD pulldown assay). These data show that mtKRAS cells have higher active KRAS compared to HKe3-wtKRAS cells. Two bands are detected as the HKe3-wtKRAS and mtKRAS cells express HA-tagged and untagged KRAS. The detection of HA-tagged KRAS is shown in the fifth lane as a positive control. The lower band shows the detection of KRAS^G13D^ (using a KRAS^G13D^-specific antibody) in the HKe3-mtKRAS cells (HA-tagged). KRAS^G13D^ is also detected in the HCT116 and HKe3 cells. **b** Total cell lysate analysis shows that total KRAS expression was higher in HKe3-mtKRAS cells compared to HKe3-wtKRAS cells. Although total MAP2K1 (MEK1) expression was similar between the four cell lines, pMAP2K1 (S217/221) was higher in mtKRAS cells indicating stronger activation of the MAPK/ERK pathway. AKT1/2 and pAKT1(T308) levels were similar in the four cell lines. **c–e** The rate of proliferation (**c**), colony formation (**d**) and wound closure (**e**) was significantly higher in HKe3-mtKRAS cells compared to HKe3-wtKRAS cells. As expected HKe3-mtKRAS cells exhibited a phenotype similar to the HCT116 cells. Error bars represent the mean ±  SD. Statistical significance was assessed using a Student’s *t*-test

To investigate the differential regulation of transcriptional responses in mtKRAS and wtKRAS cells, we performed RNAseq on two isogenic HKe3 CRC cell lines in which the genome copy of mtKRAS was disrupted and which stably express either HA-tagged KRAS^wt^ or KRAS^G13D^ using retrovirus-mediated protein expression. Additionally, RNAseq was also performed on the parental HKe3 cell line and on HCT116 cells, which express KRAS^G13D^ endogenously.^8^ The HKe3-wtKRAS and HKe3-mtKRAS cell lines have been extensively characterised previously.^7^ Both cell lines also express a genome-encoded copy of wtKRAS. Endogenous wtKRAS was expressed at similar levels in the HKe3-wtKRAS and HKe3-mtKRAS cell lines; however, the expression of mtKRAS was higher than the expression of wtKRAS protein (Fig. 1). KRAS allelic imbalance and enhanced expression of the mtKRAS protein is an important feature of KRAS-driven cancers.^23,24^ HCT116 cells also had a higher expression of mtKRAS protein.

Importantly, HKe3 cells have lost tumorigenicity and the expression of mtKRAS (but not wtKRAS) via retrovirus transfection has been shown to be required for regaining tumorigenicity.^7^ Furthermore, spheroid formation in HKe3-wtKRAS cells is not significantly different from the parental HKe3 cells, whereas HKe3-mtKRAS cells form dramatically larger spheroids, and have significantly reduced luminal apoptosis.^7^ These data confirm that it is the expression of mtKRAS which drives the oncogenic potential of the HKe3-mtKRAS cells. Consistent with their previous characterisation, we found that the HKe3-mtKRAS cells, but not the HKe3-wtKRAS cells, expressed mutant KRAS protein and these cells were characterised by increased MEK activity, increased rates of proliferation, colony formation and cell migration (Fig. 1a–e).

Whole-genome sequencing and RNA sequencing of the HKe3-wtKRAS and HKe3-mtKRAS cell lines were performed and confirmed the expected KRAS genotype in each cell line (Supplementary Table 1). We further examined these data to determine whether there were any other major genetic differences between the cell lines which could potentially drive differences in the transcriptional responses downstream of the EGFR pathway. While a large number of single-nucleotide variants (SNVs) was identified between the two cell lines, only 15 SNVs were predicted to have a high likelihood of altering protein function (Supplementary Table 1). None of the SNVs were related to EGFR signalling and the genes harbouring SNVs do not appear in a high-quality protein–protein interaction map of the EGFR signalling network, which we have recently generated (Kennedy et al., unpublished data) (Supplementary Table 1). These data confirmed that the HKe3-wtKRAS and HKe3-KRAS^G13D^ cells are a suitable model to investigate how the expression of mtKRAS alters the transcriptional response downstream of the EGFR.

To compare the transcriptional response of wtKRAS and mtKRAS cells, before and immediately after EGFR pathway activation, RNAseq was performed on RNA extracted from all four cell lines at 0, 15, 30, 60, 90 and 120 min after stimulation with the EGFR ligand, TGFα. The quantity and quality of the RNA was assessed, and each sample had a RIN > 9 (Supplementary Table 2). Prior to RNAseq, we also assessed the expression of *FOS* by RT-qPCR to ensure that we can observe transcriptional regulation within the chosen time points. *FOS* is a well-known transcription factor and immediate-early response gene (IEG) that is downstream of the EGFR pathway^6^ (Supplementary Fig. 1). TGFα stimulation induced the expression of *FOS* in all four cell lines including those expressing mtKRAS, but *FOS* was induced significantly more in the wtKRAS cells. In total, 72 cDNA libraries (6 time points × *n* = 3 replicates × 4 cell lines) were barcoded, pooled and sequenced. On average, >46 million reads were sequenced/sample, with >96% of reads aligning with a single best-hit to the genome (Supplementary Table 2).

### Transcriptional upregulation of genes involved in ribosome biogenesis, mRNA translation and metabolism in mtKRAS cells

**Fig. 2:**
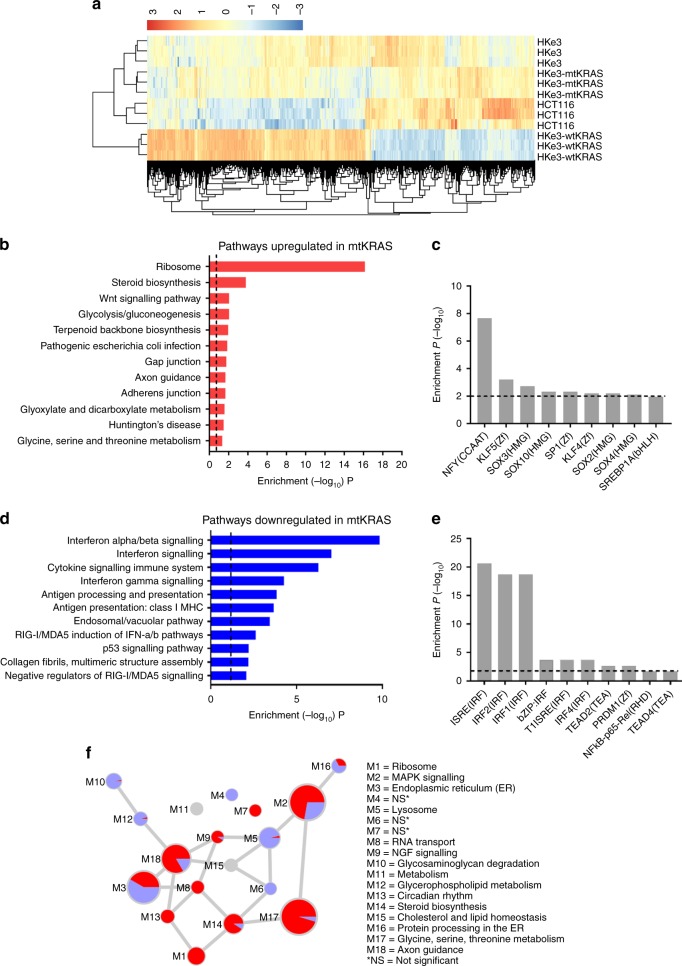
Transcriptional reprogramming of mtKRAS cells relative to wtKRAS cells. **a** Heatmap showing the expression (log2 CPM) of genes that were differentially expressed between HKe3-wtKRAS and HKe3-mtKRAS cells or HKe3-wtKRAS and HCT116 cells. The colour scale runs from blue to red representing lower to higher gene expression. **b** KEGG pathways that were significantly enriched among genes upregulated in HKe3-mtKRAS cells (in comparison to HKe3-wtKRAS cells). The dashed line represents the threshold for statistical significance at the *α* = 0.05 level. See also Supplementary Table 4. **c** Significantly enriched transcription factor-binding sites (TFBSs) in the promoters of genes upregulated in HKe3-mtKRAS cells. **d** Pathways that were significantly enriched among genes downregulated in HKe3-mtKRAS cells (in comparison to HKe3-wtKRAS cells). **e** Significantly enriched TFBSs in the promoters of genes downregulated in mtKRAS cells. **f** Network of modules identified using WGCNA showing the proportion of HKe3-mtKRAS upregulated (red) and downregulated (blue) genes mapping to each module. Only DE genes are shown. Each module is represented as a node in the module–module network, where node size is proportional to the number of genes assigned to that module. Edges represent significant co-expression between genes in different modules. Modules are annotated based on the most enriched KEGG pathways or GO terms. NS = module was not enriched for any KEGG/GO term

RNA sequencing revealed substantial differences in the transcriptome of HKe3-wtKRAS and mtKRAS cells (HKe3-mtKRAS and HCT116) prior to TGFα stimulation (Fig. 2a). More than 6000 genes were identified as significantly differentially expressed (DE) between HKe3-wtKRAS and HKe3-mtKRAS cells (Supplementary Table 3). A similar number of DE genes were identified in the HKe3 vs HCT116 comparison, and 2778 genes were identified as DE in both comparisons (Supplementary Table 3). Pathway analysis revealed that processes such as ribosome biogenesis, mRNA translation, the regulation of gene expression and metabolism were significantly enriched among HKe3-mtKRAS upregulated genes (Fig. 2b and Supplementary Table 4). Very similar processes and pathways were also enriched among genes upregulated in HCT116 cells in comparison to HKe3-wtKRAS cells (Supplementary Table 5). Given that HCT116 cells endogenously express the KRAS^G13D^ mutant at a lower level than its exogenous expression in the HKe3-mtKRAS cells, these data indicate that the transcriptional changes we observed in the HKe3-mtKRAS cells are not an artefact of the exogenous over-expression of the KRAS^G13D^ mutant. Interestingly, we observed several pathways that were upregulated in HCT116 or HKe3-mtKRAS cells compared to HKe3-wtKRAS cells, which were not significantly upregulated when compared to the parental HKe3 cell line (Supplementary Table 6). We have recently reported that the HKe3 parental cell line is not KRAS wild-type as originally thought, but still expresses some KRAS^G13D^ mutant protein.^9^ We confirmed the expression of KRAS^G13D^ mutant protein in HKe3 cells again in this study (Fig. 1a). Consistent with this, hierarchical clustering analysis of the transcriptomics data revealed that the parental HKe3 cells clustered with the other cell lines expressing KRAS^G13D^ (HCT116 and HKe3-mtKRAS) (Fig. 2a). Given this, we focused our subsequent analyses on comparing two mtKRAS cell lines (HKe3-mtKRAS and HCT116) to the HKe3-wtKRAS cells, which we have confirmed do not express any KRAS^G13D^ (Fig. 1a).

The upregulation of ribosomal protein genes was particularly evident in both HKe3-mtKRAS and HCT116 cells (Supplementary Fig. 2A). Proliferating cells are highly metabolically active and depend on ribosome biogenesis to meet their demands for faster protein synthesis^25^ and a high rate of synthesis of rRNA (as we observed in our data) is increasingly recognised as a hallmark of malignant cells.^26^ Consistent with these data, mtKRAS cells (HKe3-mtKRAS and HCT116) have much higher proliferation rates compared to the wtKRAS cells (Fig. 1c and ref. ^7^). Other upregulated pathways included processes related to cell migration and the Wnt/β-catenin signalling pathway, which is known to play a prominent role in CRC^27^ (Supplementary Fig. 2B). TFBS analysis identified that the promoters of genes preferentially upregulated in HKe3-mtKRAS cells were highly enriched for nuclear transcription factor Y (NFY)-binding sites (Fig. 2c). NFY binds to the CCAAT box, an element that has frequently been found to be enriched in promoters of genes overexpressed in tumours and to regulate metabolic pathways altered in cancer.^28^ Interestingly, despite the similar pathways that were upregulated in HCT116 and HKe3-mtKRAS cells, HCT116 upregulated genes were enriched for MYC-binding sites, not NFY-binding sites (data not shown).

Genes downregulated in HKe3-mtKRAS cells were enriched for roles in immune-related processes, most predominantly, the type I interferon signalling pathway (FDR < 1.5^E-10^) (Fig. 2d). Very similar processes were again found to be downregulated in HCT116 cells (Supplementary Table 5), confirming that this transcriptional signature is not an artefact of the over-expression of mtKRAS by retroviral transfection. Consistent with these data, TFBS analysis identified that the promoters of genes preferentially downregulated in HKe3-mtKRAS and HCT116 cells were enriched for the interferon-stimulated response element motif (Fig. 2e), a key motif in the promoters of STAT1/2-regulated genes.^29^ Another important immune-related pathway that was downregulated in both the HKe3-mtKRAS and HCT116 cells is the antigen processing and presentation pathway (Supplementary Fig. 3A). Oncogenic activation of RAS is well known to reduce the surface expression of antigen-presenting major histocompatibility complexes (MHC) on cancer cells, and such downregulation enables the cells to evade the immune response.^30^ Other downregulated pathways included the p53 signalling pathway and genes encoding cell adhesion molecules (Supplementary Fig. 3B, C). These results are consistent with previous observations that mutant RAS can induce p53 protein degradation and downregulate cellular adhesion to substrates.^31,32^

We also explored gene expression differences in HKe3-wtKRAS and mtKRAS cells using weighted gene co-expression network analysis (WGCNA).^21^ WGCNA was used to construct a co-expression network consisting of 1,143,881 interactions among 5179 genes. We identified 18 major modules of co-expressed genes in this network (Fig. 2f). Overlaying DE genes onto these modules highlighted similar pathways and processes as being differentially regulated in HKe3-mtKRAS cells as uncovered in the canonical pathway analysis. For example, there was a high proportion of HKe3-mtKRAS upregulated genes within the “ribosome” (M1) and “metabolism” modules (M12, M14 and M17).

### mtKRAS cells have enhanced expression of genes involved in metabolism

**Fig. 3:**
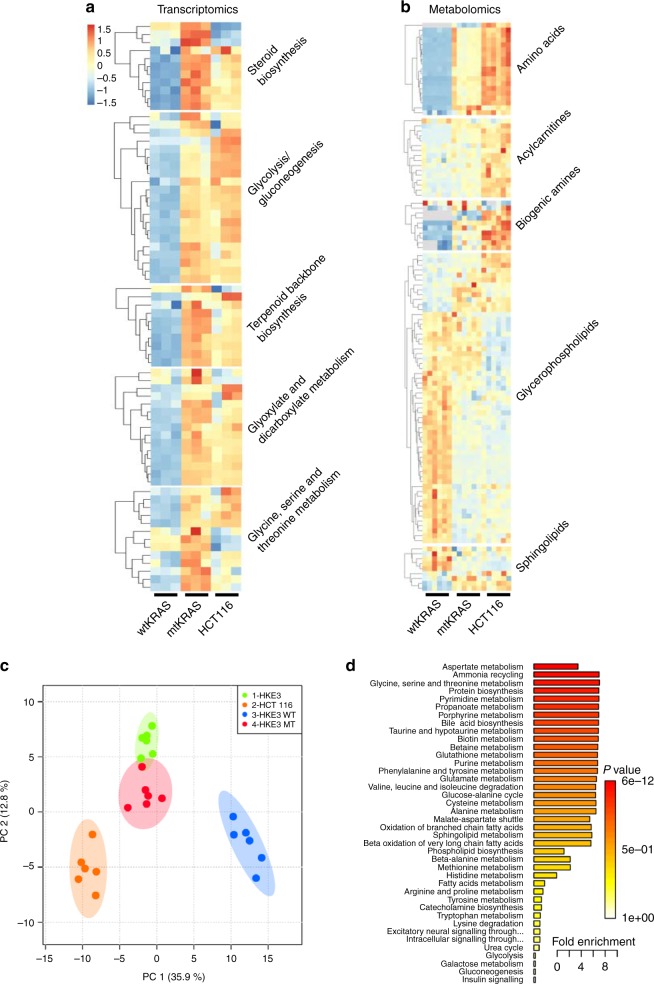
Metabolic reprogramming of mtKRAS cells. **a** Heatmap showing upregulated (red) KEGG metabolic pathways in HKe3-mtKRAS and HCT116 cells compared to HKe3-wtKRAS. The colour scale runs from blue to red representing lower to higher gene expression. **b** Heatmap showing the metabolite classes that were found to be significantly differentially abundant in HKe3-mtKRAS or HCT116 cells relative to HKe3-wtKRAS cells. **c** Principal component analysis (PCA) of targeted metabolomics data from HKe3-wtKRAS, HKe3-mtKRAS, HKe3, and HCT116 cells. **d** Enriched pathways among metabolites that are significantly more abundant in HKe3-mtKRAS cells

Both pathway analysis and WGNCA highlighted that genes upregulated in HKe3-mtKRAS cells were highly enriched for roles in diverse metabolic pathways. These pathways included steroid biosynthesis, glycolysis/gluconeogenesis, terpenoid backbone biosynthesis (which feeds into the steroid biosynthesis pathway), glyoxylate and dicarboxylate metabolism, and glycine, serine and threonine metabolism (Fig. 3a). A well-recognised hallmark of cancer cells is a switch in metabolism from oxidative phosphorylation to glycolysis, known as the Warburg effect.^33^ We observed significantly enhanced transcription of glycolysis genes in HKe3-mtKRAS and HCT116 cells compared to HKe3-wtKRAS cells (Fig. 3a and Supplementary Fig. 4A), suggesting that in CRC cells, the expression of oncogenic KRAS is sufficient to drive this metabolic switch.

The steroid biosynthesis pathway was also significantly upregulated in HKe3-mtKRAS and HCT116 cells (Fig. 3a and Supplementary Fig. 4B). The final product of steroid biosynthesis is cholesterol and oncogene-transformed cancer cells (including KRAS) require elevated levels of cholesterol to support their rapid growth.^34,35^ Targeting the C4-demethylating genes in the steroid biosynthesis pathway has been shown to sensitise previously resistant cancer cells to the EGFR inhibitors, erlotinib and cetuximab.^35,36^ Our RNAseq results provide further evidence that oncogenic KRAS CRC cells have an increased dependency on cholesterol biosynthesis.

### Targeted metabolomics analysis confirms that mtKRAS cells have substantially altered metabolism

To determine whether the metabolic differences between the HKe3-mtKRAS and HKe3-wtKRAS cells, which were predicted by the transcriptomics data, were reflected at a metabolite level, we performed a targeted metabolomics analysis of 188 endogenous metabolites in all four cell lines and found that there were significant changes in 113 (97 after Bonferroni correction) of the metabolites assessed in at least one of the cell lines (Fig. 3b and Supplementary Table 7). Principal component analysis of the quantified metabolites showed a clear separation between the metabolomic profiles of the HKe3-wtKRAS and HKe3-mtKRAS cells (Fig. 3c). Consistent with our finding of KRAS^G13D^ expression in HKe3 cells, these cells clustered with the HKe3-mtKRAS cells.

One of the most striking signatures evident in the metabolomics data was the increased abundance of almost all amino acids in the HKe3-mtKRAS and HCT116 cells (Fig. 3b, d). These results are consistent with recently published data showing that amino acids account for the majority of the carbon mass in proliferating mammalian cells.^37^ These data are also consistent with the higher rate of proliferation observed for HKe3-mtKRAS and HCT116 cells (Fig. 1c). Amino acid metabolism is also tightly linked to glycolysis,^38^ which was significantly upregulated at the transcriptional level in the HKe3-mtKRAS and HCT116 cells. The glycolytic intermediate, 3-phosphoglycerate, can be converted to serine and glycine; consistent with the predicted enhanced glycolytic activity of the HKe3-mtKRAS cells, these two amino acids were significantly more abundant in these cells. Our transcriptomics data also showed that phosphoglycerate dehydrogenase (*PHGDH*) gene expression were enhanced in HKe3-mtKRAS cells. PHGDG catalyses the transition of 3-phosphoglycerate into 3-phosphohydroxypyruvate, a crucial step in the l-serine biosynthesis pathway. Pyruvate, another glycolytic intermediate, is converted to alanine. Our targeted metabolic analysis revealed an increase of up to 10-fold in alanine concentration in the HKe3-mtKRAS cells. *SLC1A5*, which encodes an alanine transporter, was also upregulated in the HKe3-mtKRAS cells. Taken together, these data strongly support the increased activation of the glycolytic pathway in the HKe3-mtKRAS and HCT116 cells, as predicted by the transcriptomics data.

Biogenic amines were also increased in HKe3-mtKRAS and HCT116 cells, with the polyamines, spermine, and putrescine, showing the greatest increases. There is currently considerable interest in targeting polyamine metabolism in cancer.^39^ Consistent with the increase of these polyamines was the increased expression of ornithine decarboxylase 1 (*ODC1*) and spermidine/spermine N1-acetyltransferase 1(*SAT1*), which encode key enzymes in the polyamine metabolism pathway. In contrast, phosphatidylcholines (PC) tended to be decreased in HKe3-mtKRAS and HCT116 cells. PC are key components of fatty acids, lipid metabolism, signalling and cell membrane architecture, and there is a growing interest in targeting lipid metabolism in cancer.^40^ Several acylcarnitines were also found to be significantly altered in the HKe3-mtKRAS and HCT116 cells. Acylcarnitines with long chains (C8–C18) were slightly depleted in the HKe3-mtKRAS cells, while the short-to-medium chains (C2–C5) and free carnitine (C0) were increased. Carnitine and its esters are associated with enhanced mitochondrial fatty acid β-oxidation which may help support the increased energy demands of mtKRAS cells.^41^ In summary, our targeted metabolomics analysis strongly supports the conclusions of our transcriptional analysis that mtKRAS cells are substantially metabolically reprogrammed.

### mtKRAS CRC cells are highly transcriptionally responsive to signalling through the EGFR

**Fig. 4:**
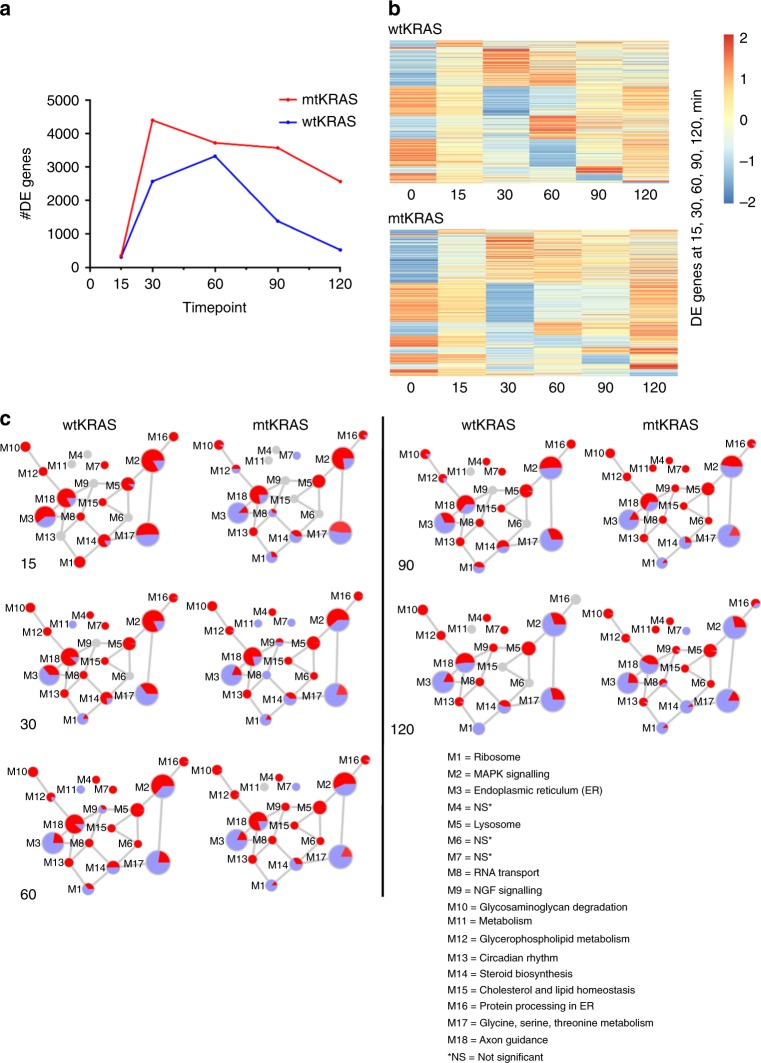
HKe3-mtKRAS cells are highly transcriptionally responsive to further activation of the EGFR pathway via TGFα stimulation. **a** Number of differentially expressed genes at 15, 30, 60, 90 and 120 min following TGFα stimulation (relative to unstimulated) in HKe3-wtKRAS, HKe3-mtKRAS, HKe3 and HCT116 cells. **b** Heatmaps showing expression levels of DE genes post-TGFα stimulation in HKe3-wtKRAS and HKe3-mtKRAS cells. Gene expression at 0 min (prior to stimulation) is shown for comparison. The heatmaps reveal distinct “blocks” of genes that are up- or downregulated at the different time points, revealing unique temporal waves of transcription post-stimulation in both cell lines. Similar data for HKe3 and HCT116 cells not shown. **c** Network of modules identified using WGCNA showing the proportion of HKe3-mtKRAS upregulated (red) and downregulated (blue) genes mapping to each module at each time point. Only DE genes are shown. Each module is represented as a node in the module-module network, where node size is proportional to the number of genes assigned to that module. Edges represent significant co-expression between genes in different modules. Modules are annotated based on the most enriched KEGG pathways or GO terms. NS = module was not enriched for any KEGG/GO term

The transcriptional response of HKe3-mtKRAS and wtKRAS cells following TGFα stimulation followed a broadly similar pattern, with relatively unique temporal waves of transcription that peaked between 30 and 60 min following stimulation (Fig. 4a, b and Supplementary Table 8). Interestingly, while HCT116 cells also mounted a substantial transcriptional response to TGFα stimulation, this response was considerably more muted in terms of numbers of DE genes compared with the other three cell lines. In HKe3-mtKRAS cells, the transcriptional response to TGFα stimulation peaked at 30 min, with >4,000 genes identified as DE (FDR < 0.05). Interestingly, this was nearly 1500 more genes than were identified in the HKe3-wtKRAS cells. The peak transcriptional response (defined by the number of DE genes) in the HKe3-wtKRAS cells was also later at 60 min. The response in HKe3-mtKRAS cells was also more sustained with >2500 genes DE at 120 min after stimulation, in comparison to ~500 DE genes in the wtKRAS cells at this time point. Our data show that oncogenic KRAS CRC cells are highly transcriptionally responsive to activation of the EGFR via TGFα stimulation, which is surprising as mtKRAS is thought to decouple downstream signalling from EGFR stimulation.

### Pathway and network analysis of genes upregulated in mtKRAS and wtKRAS cells post-TGF-α stimulation

To profile which pathways and processes were differentially regulated in wtKRAS and mtKRAS cells following stimulation, we employed canonical pathway analysis and WGCNA. Analysis of upregulated genes revealed that both HKe3-wtKRAS and HKe3-mtKRAS cells induced the expression of genes involved in pathways, such as the AP-1 transcription factor network and MAPK signalling, that are well known to be downstream of the EGFR (Fig. 4c and Supplementary Table 9). This was also observed in HCT116 cells. The AP-1 transcription factor network includes many key transcription factors that are well known to be activated rapidly in response to engagement of the EGFR pathway and have been termed “immediate-early genes” (IEGs).^6^ Upregulated genes included the AP-1 complex components, *FOS, FOSL1* and *JUNB*, and genes encoding the zinc-finger transcription factors EGR1, EGR2 and EGR3 (Supplementary Fig. 5A, B).

Network analysis also highlighted several differences in the upregulation of certain network modules after stimulation in the HKe3-wtKRAS and HKe3-mtKRAS cell lines (Fig. 4c and Supplementary Table 10**)**. At 15 min, for example, modules including the ribosome module (M1) and the steroid biosynthesis module (M14) had a higher proportion of HKe3-wtKRAS upregulated genes (relative to unstimulated cells) than mtKRAS cells. Given that these processes were already highly transcriptionally active in the HKe3-mtKRAS cells prior to TGFα stimulation, this may largely reflect the HKe3-wtKRAS cells “catching up” with the HKe3-mtKRAS cells after stimulation, though it is interesting that further activation of the pathway does not appear to elicit enhanced upregulation of genes involved in these processes in the HKe3-mtKRAS cells. At 30 min, many of the modules had similar proportions of upregulated genes in both HKe3-wtKRAS and HKe3-mtKRAS cells, though the NGF signalling module (M9) was not differentially regulated in the HKe3-wtKRAS cells but was in the HKe3-mtKRAS cells. The RNA transport module (M8) showed regulation in opposite directions at this time point in the two cell lines. The NGF signalling module (M9) also had a higher proportion of upregulated genes in HKe3-mtKRAS cells at 60 min, but most other modules showed similar proportions of upregulated genes in both cell lines. At 90 and 120 min, HKe3-mtKRAS cells preferentially upregulated several modules related to metabolism (M11, M14, M17).

### TGFα stimulation of mtKRAS cells leads to the downregulation of genes involved in protein synthesis

**Fig. 5:**
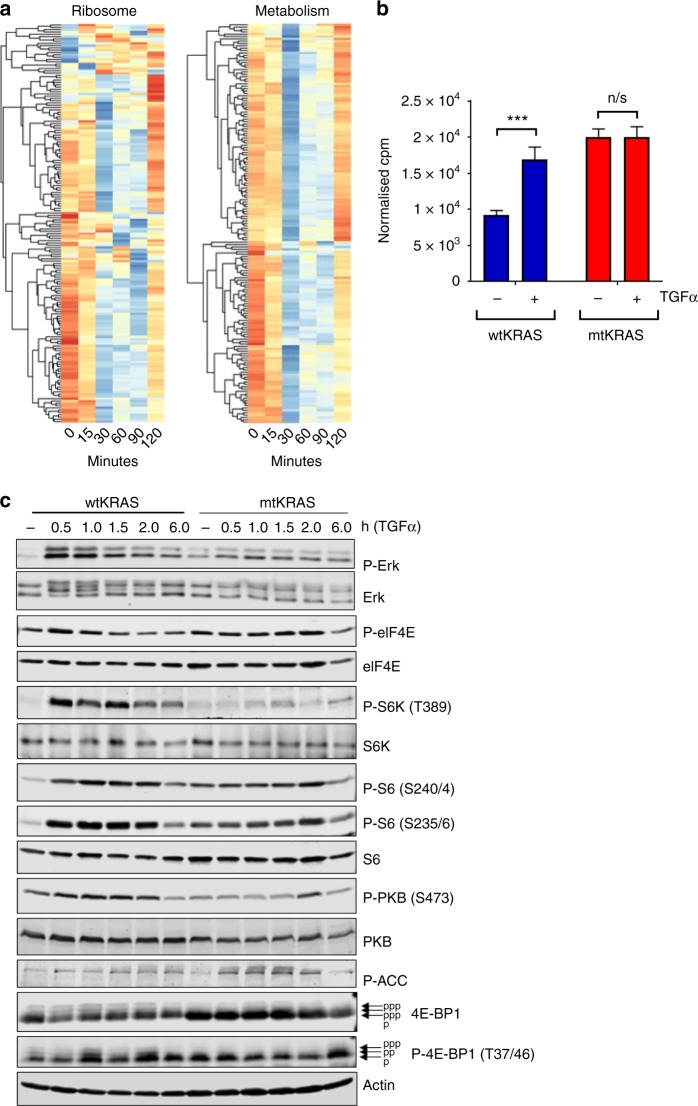
Deficient activation of protein synthesis in HKe3-mtKRAS cells after TGFα stimulation. **a** Heatmap showing the transcriptional response of genes involved in the KEGG “ribosome” and “metabolism” pathways at 0, 15, 30, 60, 90 and 120 min after stimulation with TGFα in HKe3-mtKRAS cells. **b** The rate of protein synthesis, as assessed by the incorporation of [^35^S]methionine into HKe3-wtKRAS and HKe3-mtKRAS cells, with or without stimulation by TGFα. Cpm values were normalised to concentrations of intracellular methionine. **c** HKe3-wtKRAS and HKe3-mtKRAS cells were starved of serum for 18 h and then stimulated with 0.01 μg/mL TGFα for the indicated periods of time. Cells were then lysed, and lysates were subjected to SDS-PAGE and immunoblotting analysis using the indicated phospho- (P-) or total proteins. The arrows next to the images for 4E-BP1 indicate the differentially phosphorylated forms of 4E-BP1 (also shown by p, pp; note that more heavily phosphorylated forms of 4E-BP1 migrate more slowly)

Activation of the EGFR signalling pathway drives cell growth, proliferation, survival and metastasis and as mentioned above, ribosome biogenesis is a major process required to meet the demands of increased protein synthesis in proliferating cells.^25^ Surprisingly then, TGFα stimulation of all four CRC cell lines was associated with a pronounced but transient downregulation of genes involved in a range of processes related to ribosome biogenesis, mRNA translation and metabolism (Fig. 5a and Supplementary Fig. 6). These data suggest that treating these cells with TGFα leads to an unexpected transient inhibition of the transcription of genes that encode proteins involved in protein synthesis. By 120 min these processes were no longer significantly downregulated. Interestingly, this repression appeared to be more transient in the HKe3-wtKRAS and HKe3 cells (Supplementary Fig. 6 and Supplementary Table 11). To investigate the impact of mutant KRAS on protein synthesis, we used the incorporation of [^35^S]methionine to measure de novo protein synthesis in HKe3-wtKRAS and HKe3-mtKRAS cells in response to stimulation by TGFα. As expected, the basal rate of protein synthesis was significantly higher in mtKRAS cells than in wtKRAS cells (Fig. 5b). These data are consistent with our transcriptomics data (which show significantly increased basal expression of genes encoding ribosomal proteins in the mtKRAS cells) and our cell proliferation data (showing faster proliferation of the mtKRAS cells). Interestingly, however, while TGFα treatment strongly activated protein synthesis in wtKRAS cells, it did not further activate protein synthesis above the already higher basal levels in the mtKRAS cells (Fig. 5b).

### TGFα stimulation leads to the activation of mTOR signalling in wtKRAS but not mtKRAS cells

We next considered which signalling pathways might be involved in the regulation of protein synthesis in mtKRAS cells in response to TGFα. Hke3-wtKRAS showed low basal pERK which was rapidly increased following stimulation with TGFα (Fig. 5c and Supplementary Fig. 7). As expected, the KRAS^G13D^ constitutively active mutant cells had higher basal levels of pERK (indicating constitutive activation of ERK signalling) than wtKRAS cells; however, a modest increase in pERK levels was evident in the mtKRAS cells in response to TGFα (Fig. 5c), indicating that despite the constitutive activation, this pathway is responsive to exogenous EGFR ligands. These data are consistent with our transcriptomics data which showed, following TGFα stimulation, the induction of IEGs, which are well-established to be downstream of the EGFR pathway (Supplementary Fig. 5). Consistent with the data for pERK in the wtKRAS cells, a similar pattern was observed in these cells for the phosphorylation of the regulatory initiation factor eIF4E which is phosphorylated by the MNK kinases downstream of ERK.^42^ Interestingly, levels of the ribosomal protein S6 (a component of the small ribosomal subunit) were higher in mtKRAS cells, indicating increased levels of ribosomes, which is consistent with the gene expression data (Supplementary Fig. 2A) and provides an explanation for the higher basal rate of protein synthesis seen in mtKRAS cells.

To gain insight into why TGFα does not further activate protein synthesis beyond the already high basal levels in mtKRAS cells, we examined the activation of mTORC1 signalling, a major pathway controlling the protein synthesis machinery.^43^ Treating HKe3-wtKRAS cells with TGFα quickly and strongly activated mTORC1 signalling as shown by the enhanced phosphorylation of S6 kinase (Fig. 5c), a substrate for mTORC1, and its own substrate, ribosomal protein S6. In stark contrast, in HKe3-mtKRAS cells, TGFα did not appreciably stimulate phosphorylation of S6K1 above its low basal level (Fig. 5c). Similarly, phosphorylation of 4E-BP1 (as indicated by its mobility on SDS-PAGE, where phosphorylated forms migrate more slowly) was also not enhanced above its low basal levels by TGFα in mtKRAS cells.

Signalling through protein kinase B (PKB, also termed Akt, which lies downstream of PI3-kinase) stimulates mTORC1 by inactivating the tuberous sclerosis complex, TSC.^44^ In HKe3-wtKRAS cells TGFα stimulated phosphorylation of PKB/Akt, which is associated with its activation, within 30 min, but this rapid activation was severely ablated in the Hke3-mtKRAS cells (Fig. 5c). Since mTORC1 signalling is a major regulator of protein synthesis, the inability of TGFα to stimulate further protein synthesis in mtKRAS cells likely reflects the failure to switch on this key anabolic pathway.

We also noticed that the “*LKB1 signalling events*” pathway was more significantly enriched among upregulated genes in mtKRAS cells than in wtKRAS cells, at 30 and 60 min after TGFα stimulation (Supplementary Table 11 and Supplementary Fig. 8). LKB1 is a serine/threonine kinase that directly phosphorylates and activates the adenosine monophosphate-activated protein kinase (AMPK).^45^ Under conditions of metabolic stress, AMPK negatively regulates a broad spectrum of energy-consuming anabolic cellular functions including protein synthesis, growth, metabolism, and proliferation, by a range of mechanisms including repression of mTORC1 signalling.^46,47^ We found that TGFα transiently activated AMPK signalling in HKe3-mtKRAS cells (and to a less extent wtKRAS cells) at 30–60 min following stimulation, as shown by greater levels of phosphorylated acetyl-CoA carboxylase (ACC), a direct AMPK substrate (Fig. 5c). These data were consistent with our transcriptomics data which also showed the transient downregulation of genes involved in protein synthesis and metabolism at these time points in both wtKRAS and mtKRAS cells (Fig. 5a). To assess whether mtKRAS cells are more vulnerable to AMPK inhibition following TGFα stimulation than the wtKRAS cells, we made use of a recently described and specific inhibitor of AMPK (available commercially as SBI-020965^48^). Interestingly, while treatment with SBI-020965 strongly inhibited mtKRAS cell proliferation, the wtKRAS cells were equally susceptible (Supplementary Fig. 9). It thus seems likely that the inability of TGFα to stimulate protein synthesis in mtKRAS cells reflects its lack of stimulation of PKB and downstream pathways linked to protein synthesis, in particular mTORC1.

## Discussion

To investigate the reprogramming of the transcriptional networks downstream of the EGFR pathway in wild type and mutant KRAS cells, we profiled transcriptome-wide gene expression in two HKe3 CRC cell lines which had been retrovirally transfected to re-express either the G13D mutant or wild-type versions of the KRAS gene^7^ and in HCT116 cells, which endogenously express KRAS^G13D^. We found that there were >6000 genes that were DE prior to stimulation in HKe3-mtKRAS cells compared to wtKRAS cells. Genes upregulated in HKe3-mtKRAS or HCT116 cells were enriched for roles in ribosome biogenesis and mRNA translation. Ribosome biogenesis, including rRNA synthesis, is a major metabolic effort supporting cell proliferation and is recognised both as a hallmark of malignant cells and as a potential target of therapeutic intervention.^26^ In addition, many other processes related to transcription, and to translation initiation, termination and elongation were upregulated in the mtKRAS cells. Many oncogenes including KRAS are well known to affect the transcriptional machinery, making aberrant translation a widespread characteristic of tumour cells, and the expression of many proteins involved in translation is associated with poor prognosis in CRC.^49^

HKE3-mtKRAS and HCT116 cells also preferentially upregulated genes encoding proteins involved in many metabolic pathways including glycolysis/gluconeogenesis. Previous studies have confirmed that there is an association between the increased expression of oncogenic KRAS and the expression of proteins involved in glycolysis.^50^ The steroid biosynthesis pathway was also transcriptionally upregulated in the mtKRAS cell lines. Cholesterol is associated with an increased risk of CRC^51^ and targeting its synthesis has been shown to sensitise previously resistant cancer cells to the EGFR inhibitors, erlotinib and cetuximab.^35,36^ Our RNAseq results provide further confirmation that oncogenic KRAS cells have an increased dependency on cholesterol biosynthesis and targeting this pathway could thus be a therapeutic strategy in KRAS mutant CRC patients. Target metabolomics analysis confirmed the predicted metabolic reprogramming of mtKRAS cells.

Our RNAseq analysis also showed that oncogenic KRAS cells have significantly downregulated gene expression of interferon response genes, and genes involved in antigen processing and presentation, and the p53 signalling pathway. mtKRAS cells had repressed expression of key transcription factors regulating the interferon signalling pathway (STATs and IRFs). Previous studies have also confirmed this finding, demonstrating that oncogenic KRAS inhibits the expression of interferon-responsive genes through inhibition of STAT1 and STAT2 expression in colorectal cell lines.^52^ Oncogenic activation of RAS is also well known to reduce the expression of antigen-presenting MHC on cancer cells, and such downregulation results in decreased immunogenicity of the RAS-transformed cells, thus enabling the cells to evade the immune response.^30^ This finding may have implications for selecting patients who will benefit from immunotherapy.

Further investigation of the early transcriptional response of wtKRAS and mtKRAS cells to activation of the EGFR pathway revealed that HKe3-mtKRAS and, to a lesser extent, HCT116 cells mount a rapid and substantive transcriptional response to TGFα stimulation that is sustained to at least 2 h after stimulation. As the KRAS^G13D^ mutant is a constitutively active mutant, a widely-held belief is that mutant KRAS cells are not dependent on upstream EGFR signalling. Our data show that oncogenic KRAS CRC cells are still highly transcriptionally responsive to signalling through the EGFR. Our data is consistent with several other studies that have also demonstrated that oncogenic KRAS cells are dependent on upstream EGFR signalling for full activation of downstream signalling.^53–55^

It is well known that activation of the EGFR signalling pathway drives cell growth, proliferation, survival and metastasis. Remarkably, our RNAseq analysis results showed that TGFα stimulation was associated with a pronounced downregulation of genes involved in ribosome biogenesis and mRNA translation, suggesting there is an unexpected inhibition of the transcription of genes with roles in protein synthesis. This appeared to be more sustained in the HKe3-mtKRAS and HCT116 cells and we subsequently confirmed that protein synthesis was not activated beyond the already higher basal levels in the HKe3-mtKRAS cells following TGFα stimulation, whereas protein synthesis was rapidly induced in TGFα stimulated HKe3-wtKRAS cells.

One possible explanation for these data was the observed upregulation of genes involved in the LKB1 signalling pathway, which was identified as significantly enriched among upregulated genes post-stimulation. During metabolic stress, LKB1 activates AMPK to inhibit cell growth and proliferation, thereby saving cellular energy and ensuring cell survival. Consistent with this, while TGFα strongly activated anabolic mTORC1 signalling in HKe3-wtKRAS cells, such activation was severely attenuated in HKe3-mtKRAS cells. This could be due to impaired activation of PKB/AKT and/or to higher levels of AMPK signalling in the HKe3-mtKRAS cells, since AMPK can impair mTORC1 signalling.^44^ ERK signalling drives a feedback loop that impairs activation of PI 3-kinase and hence Akt.^56,57^ Since ERK signalling is, as expected, elevated in mtKRAS cells, it seems likely that TGFα cannot activate PKB/mTORC1 signalling in mtKRAS cells due to the high prevailing levels of ERK signalling. These data suggest that inhibitors of PI 3-kinase, PKB, or rapalogs that inhibit mTORC1 signalling, are unlikely to be effective in mtKRAS tumours, as these pathways are refractory to activation.

## Supplementary information

Supplementary Figure and Table Legends

Supplementary Figures

Supplementary Table 1

Supplementary Table 2

Supplementary Table 3

Supplementary Table 4

Supplementary Table 5

Supplementary Table 6

Supplementary Table 7

Supplementary Table 8

Supplementary Table 9

Supplementary Table 10

Supplementary Table 11

## Data Availability

Genome sequencing data were submitted to the NCBI sequence read archive, accession number PRJNA467910. RNAseq data are deposited in the Gene Expression Omnibus, accession number GSE105094 and GSE110649
