# Supplementary material for: Transcriptional and metabolic rewiring of colorectal cancer cells expressing the oncogenic KRASG13D mutation
Source: Br J Cancer. 2019 May 28;121(1):37–50. doi: 10.1038/s41416-019-0477-7 (PMC6738113; doi:10.1038/s41416-019-0477-7)
Supplement: Supplementary file 1 — Supplementary Figure and Table Legends [file 41416_2019_477_MOESM1_ESM.docx]

**Supplementary Figure Legends**

**Supplementary Figure 1.** RT-qPCR quantification of *FOS* gene expression in HKe3-wtKRAS, HKe3-mtKRAS, HKe3, and HCT116 cells post-stimulation with TGFα. Error bars represent the mean +/- SEM.

**Supplementary Figure 2.** Heatmaps highlighting the upregulation of **(A)** ribosomal genes, and **(B)** the Wnt signaling pathway genes in HKe3-mtKRAS and HCT116 cells. The colour scale runs from blue to red representing lower to higher gene expression.

**Supplementary Figure 3.** Heatmaps highlighting the downregulation of **(A)** the antigen processing and presentation pathway, **(B)** the p53 signaling pathway, and **(C)** cell adhesion molecule (CAM) genes, in HKe3-mtKRAS and HCT116 cells.

**Supplementary Figure 4.** Upregulation of the **(A)** glycolysis and **(B)** steroid biosynthesis KEGG pathways in HKe3-mtKRAS and HCT116 cells. The colour scale runs from blue to red representing lower to higher gene expression. Upregulated genes are highlighted in red on the KEGG pathway diagrams.

**Supplementary Figure 5.** The transcriptional response of selected transcription factors downstream of the EGFR pathway after stimulation of **(A)** HKe3-wtKRAS or **(B)** HKe3-mtKRAS cells with TGFα. The gene expression data is from RNA sequencing. Log_2_ read counts per million (CPM) are shown. The colour scale runs from blue to red representing lower to higher gene expression.

**Supplementary Figure 6. (A)** The transcriptional response of KEGG ribosomal pathway genes after stimulation of HKe3-mtKRAS, HKe3-wtKRAS, HKe3 and HCT116 cells with TGFα. **(B)** The transcriptional response of KEGG metabolism genes after stimulation of HKe3-mtKRAS, HKe3-wtKRAS, HKe3 and HCT116 cells with TGFα. The gene expression data is from RNA sequencing. Log_2_ read counts per million (CPM) are shown. Only genes that were differentially expressed at at least one timepoint are shown. The colour scale runs from blue to red representing lower to higher gene expression.

**Supplementary Figure 7.** Quantification of Western blot data shown in Figure 5C.

**Supplementary Figure 8.** Gene expression profiles of genes within the LKB1-AMPK pathway at 0, 15, 30, 60, 90 and 120 min post-stimulation with TGFα in HKe3-wtKRAS (blue) and HKe3-mtKRAS (red) cells. The gene expression data is from RNA sequencing. Read counts per million (CPM) are shown.

**Supplementary Figure 9.** The AMPK inhibitor SBI-020965 inhibited cell proliferation in **(A)** HKe3-wtKRAS and **(B)** HKe3-mtKRAS cells. Stimulation with TGFα (1 ng) was unable to rescue the inhibition of cell proliferation by the inhibitor. Error bars represent the mean +/- SEM. Statistical significance was assessed using the two-tailed Student’s t test.

**Supplementary Tables**

**Supplementary Table 1.** Authentication of the KRAS genotype in both cell lines. The HKe3-wtKRAS cell line is homozygous KRAS wild-type, while the HKe3-mtKRAS cell line correctly is heterozygous KRAS wild-type/G13D, as expected. All RNA-level SNVs that differ between the HKe3-wtKRAS and mtKRAS cells. All DNA-level SNVs that differ between the HKe3-wtKRAS and mtKRAS cells. Matching of high-impact DNA and RNA-level SNVs to the EGFR PPI network (Kennedy *et al.,* in review) showed that none of the high-impact SNVs map to the proteins in the EGFR network.

**Supplementary Table 2.** QC metrics for the 72 RNAseq samples.

**Supplementary Table 3.** Differentially expressed genes (FDR ≤ 0.05) identified between HKe3-wtKRAS and HKe3-mtKRAS cells; HKe3-wtKRAS and HCT116 cells; or HKe3 and HCT116 cells; prior to TGFα stimulation.

**Supplementary Table 4.** Pathway analysis of DE genes identified between HKe3-mtKRAS and wtKRAS cells prior to TGFα stimulation.

**Supplementary Table 5.** Pathway analysis of DE genes identified between HCT116 and HKe3-wtKRAS cells prior to TGFα stimulation.

**Supplementary Table 6.** Pathway analysis of DE genes identified between HCT116 and HKe3 parental cells prior to TGFα stimulation.

**Supplementary Table 7.** Targeted metabolomics analysis of the HKe3-mtKRAS, HKe3-wtKRAS, HKe3 and HCT116 cell lines.

**Supplementary Table 8.** Differentially expressed genes (FDR ≤ 0.05) at 15, 30, 60, 90, and 120 minutes post-stimulation with TGFα (relative to timepoint 0) in the HKe3-mtKRAS, HKe3-wtKRAS, HKe3 and HCT116 cell lines.

**Supplementary Table 9.** Upregulated pathways in HKe3-mtKRAS, HKe3-wtKRAS and HCT116 cells at 15, 30, 60, 90, and 120 minutes post-stimulation with TGFα (relative to timepoint 0).

**Supplementary Table 10.** Weighted Co-expression Network Analysis (WGCNA). Modules identified by the WGCNA R package; Proportion of up- and downregulated genes in each module prior and post-stimulation with TGFα; Module Annotation.

**Supplementary Table 11.** Downregulated pathways in HKe3-mtKRAS, HKe3-wtKRAS and HCT116 cells at 15, 30, 60, 90, and 120 minutes post-stimulation with TGFα (relative to timepoint 0).
