# Supplementary material for: Transcriptional and metabolic rewiring of colorectal cancer cells expressing the oncogenic KRASG13D mutation
Source: Br J Cancer. 2019 May 28;121(1):37–50. doi: 10.1038/s41416-019-0477-7 (PMC6738113; doi:10.1038/s41416-019-0477-7)

Supplementary Figure 1

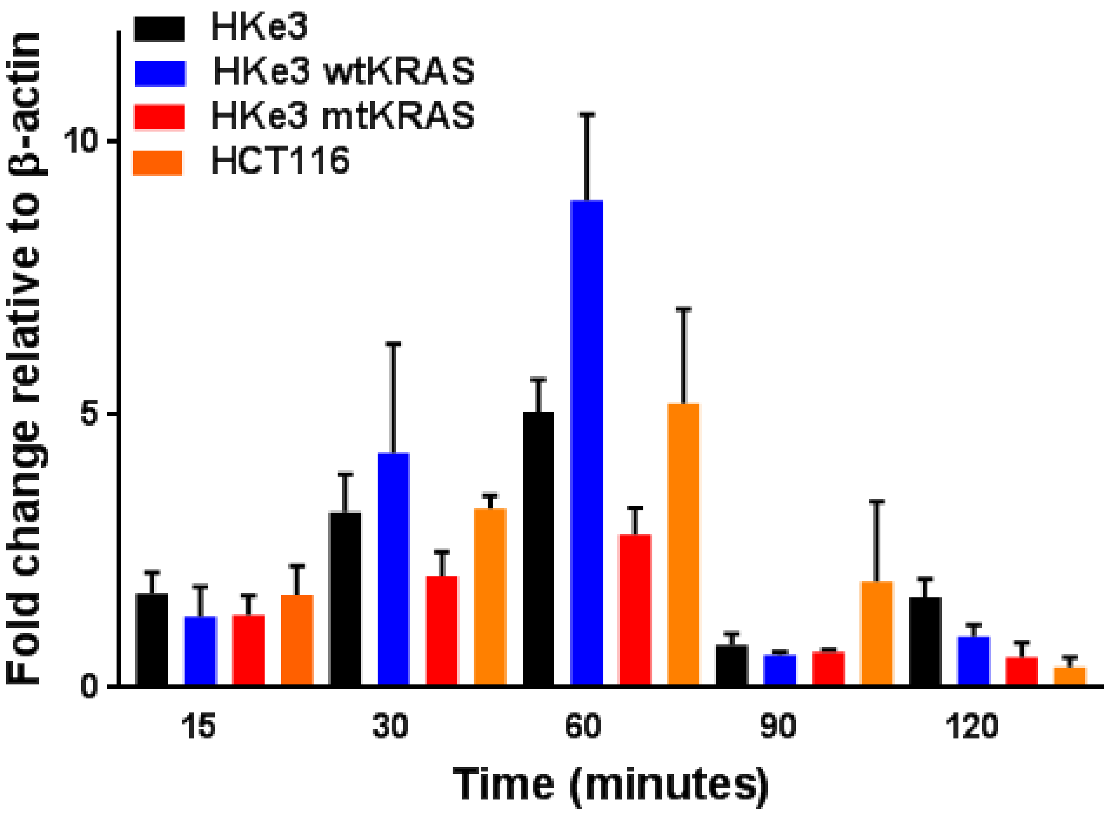

Supplementary Figure 2

A

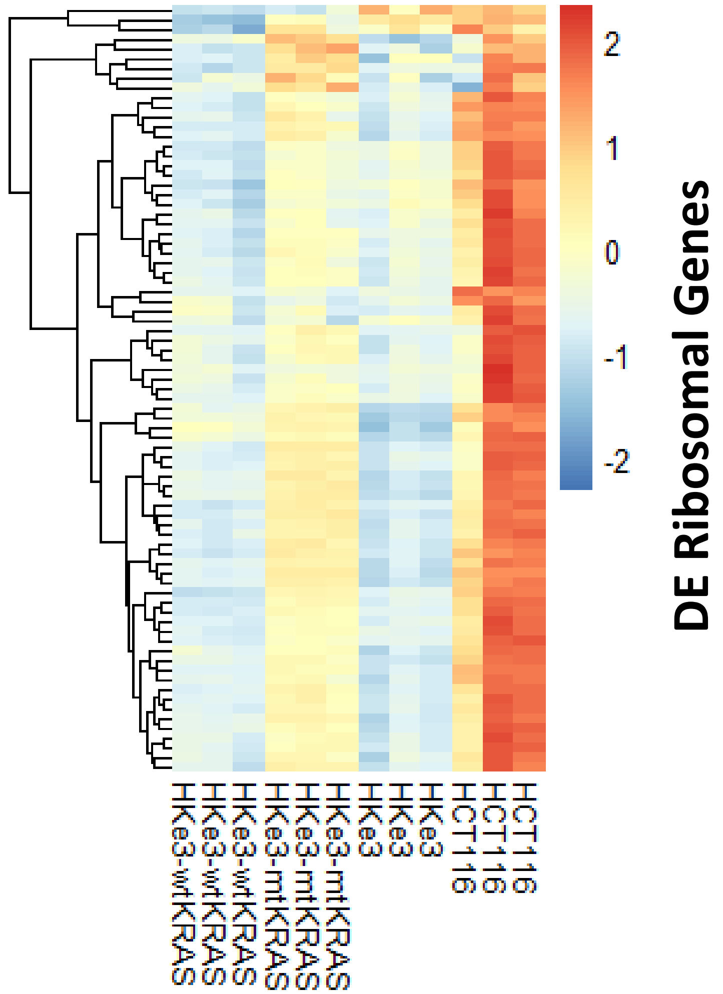

B

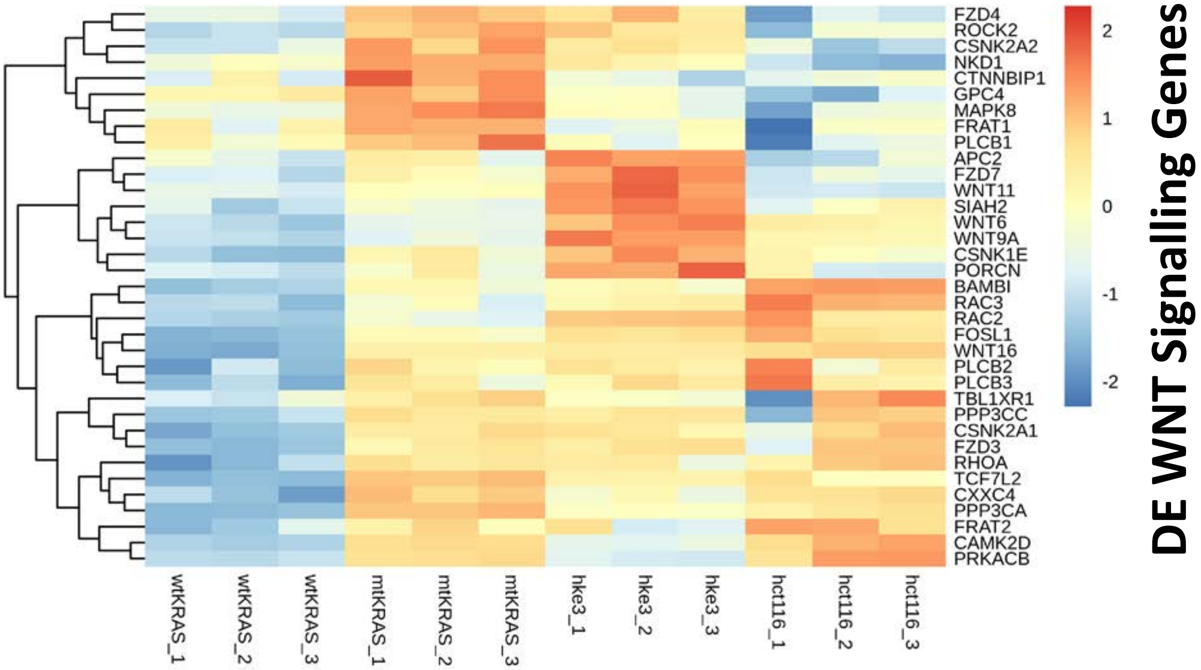

Supplementary Figure 3

A

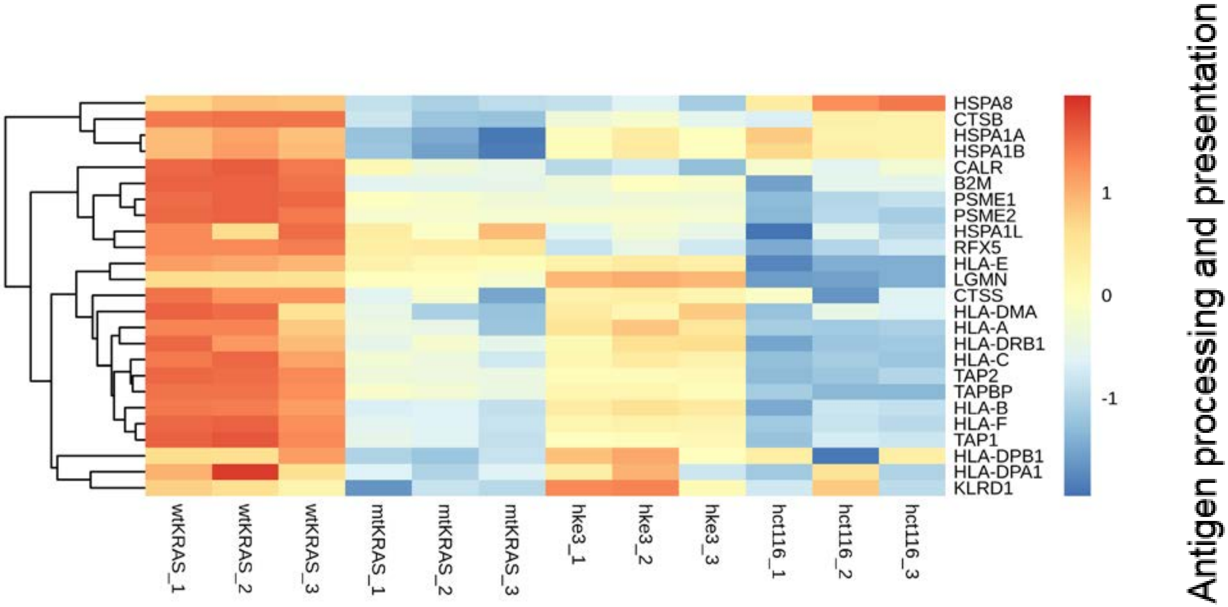

B

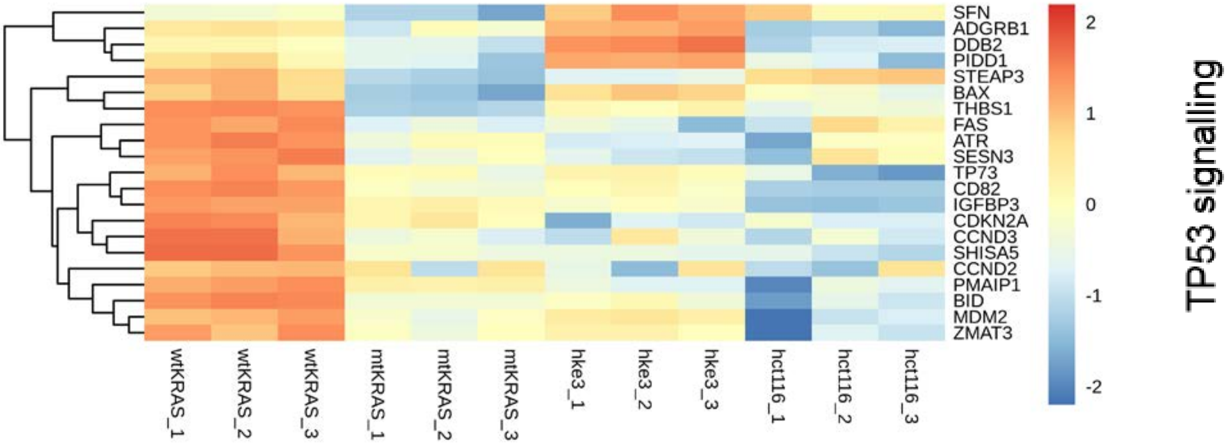

C

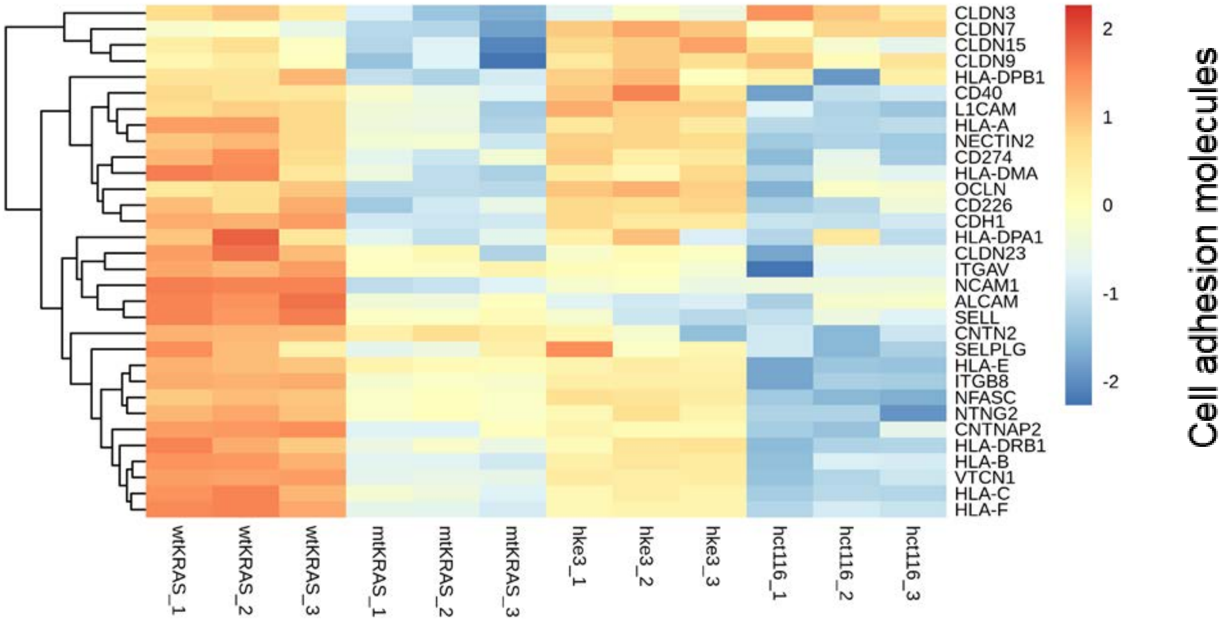

Supplementary Figure 4

A

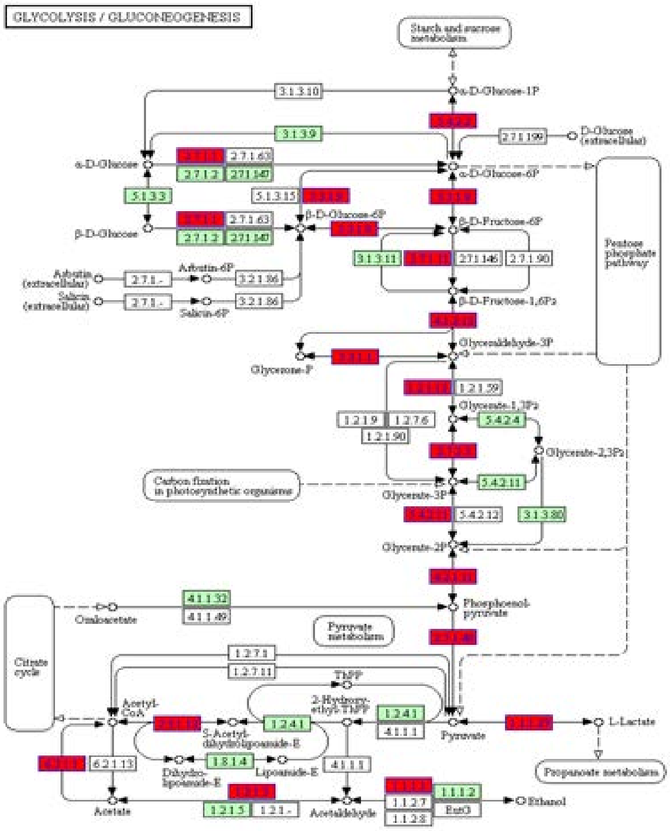

DE Glycolysis Genes

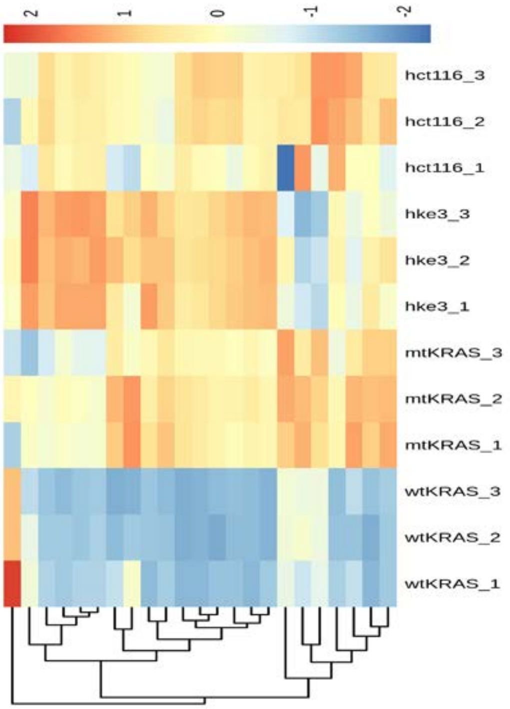

DE Steroid Biosynthesis Genes

B

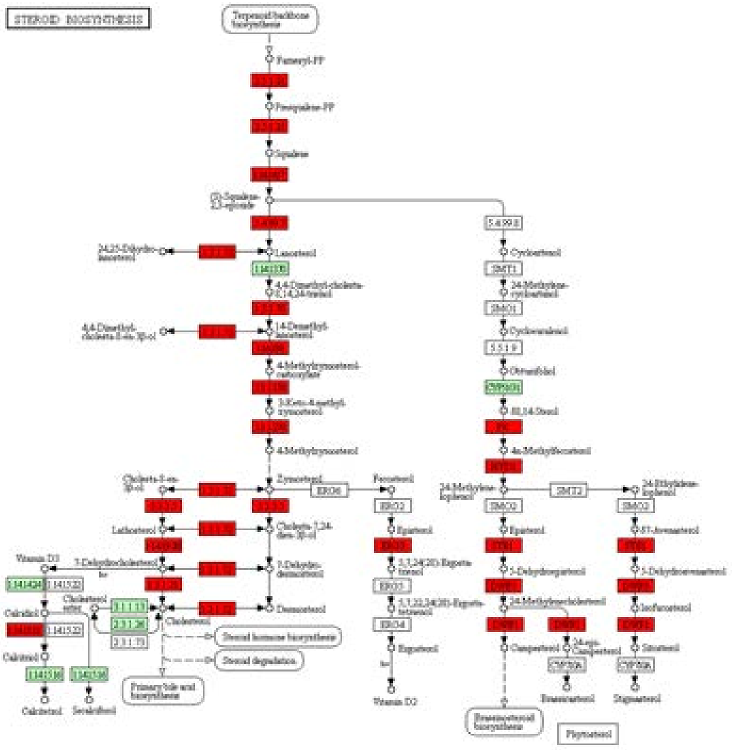

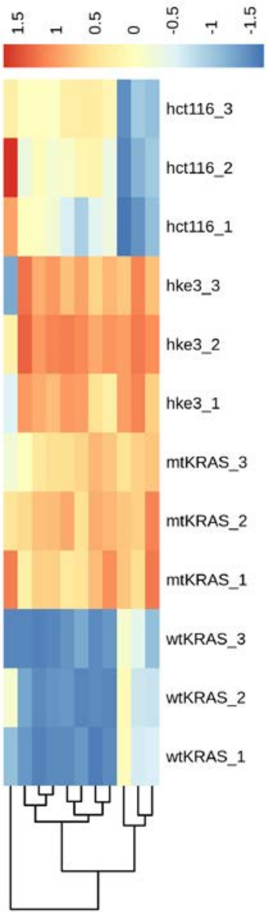

Supplementary Figure 5

A

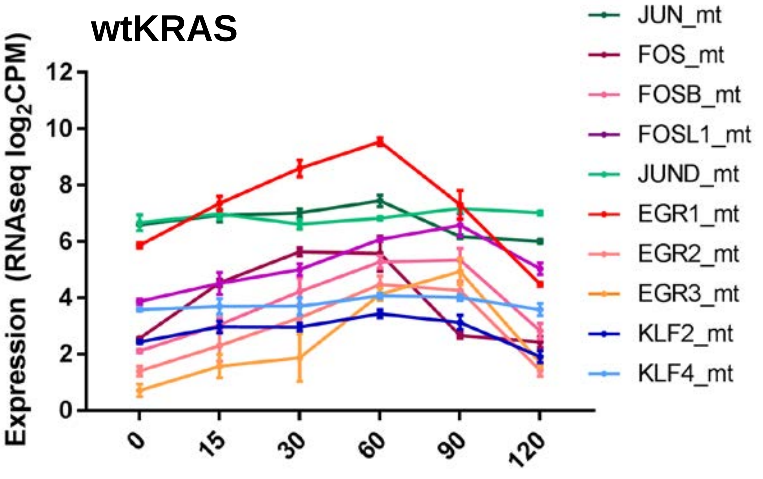

B

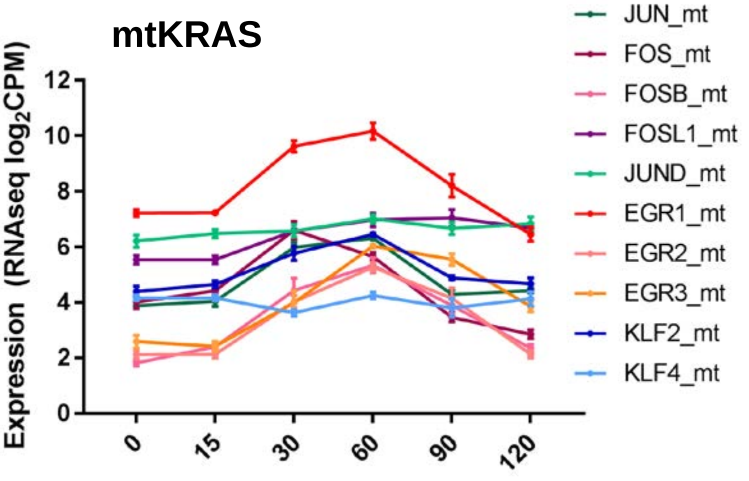

Supplementary Figure 6

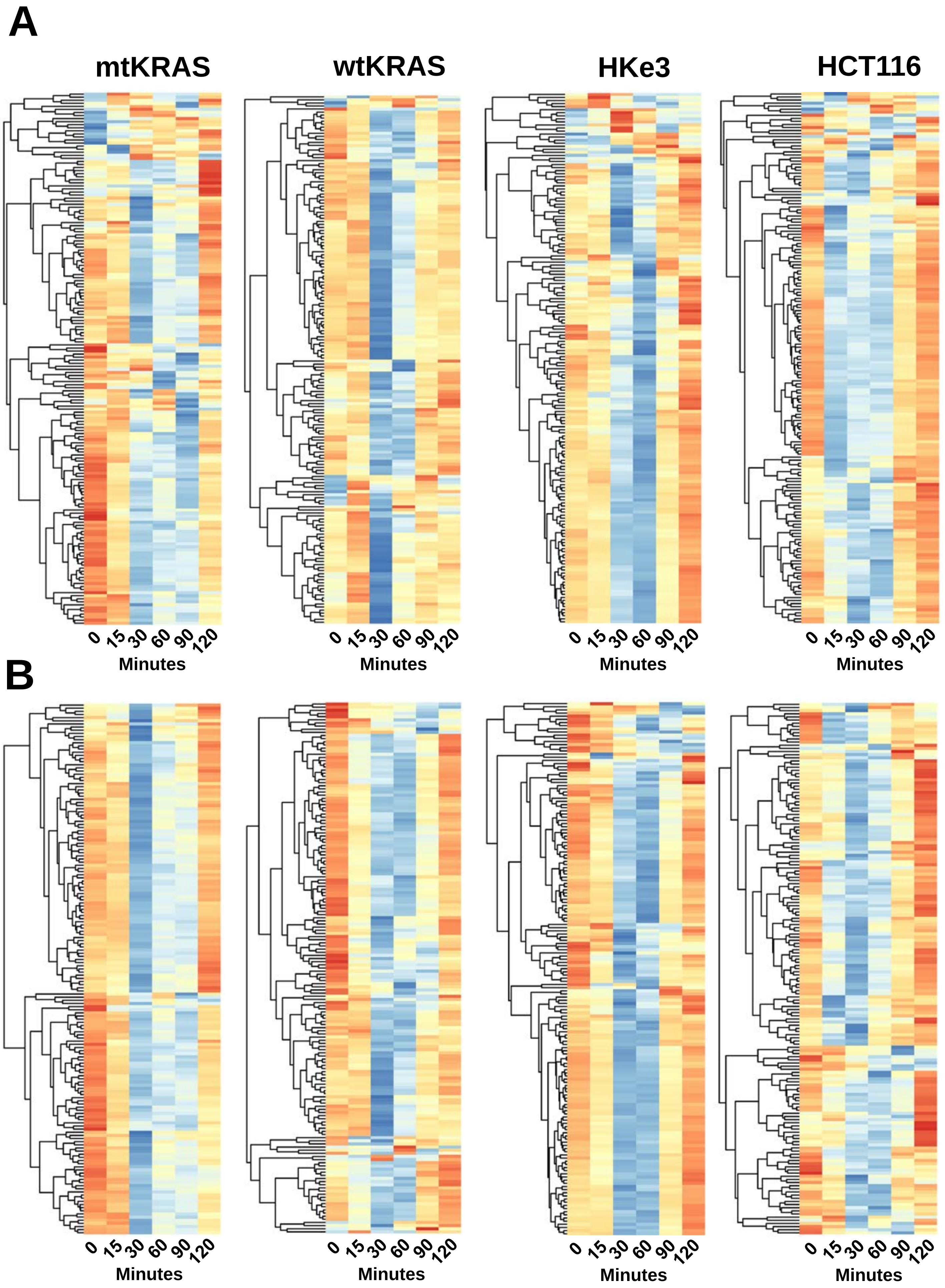

Supplementary Figure 7

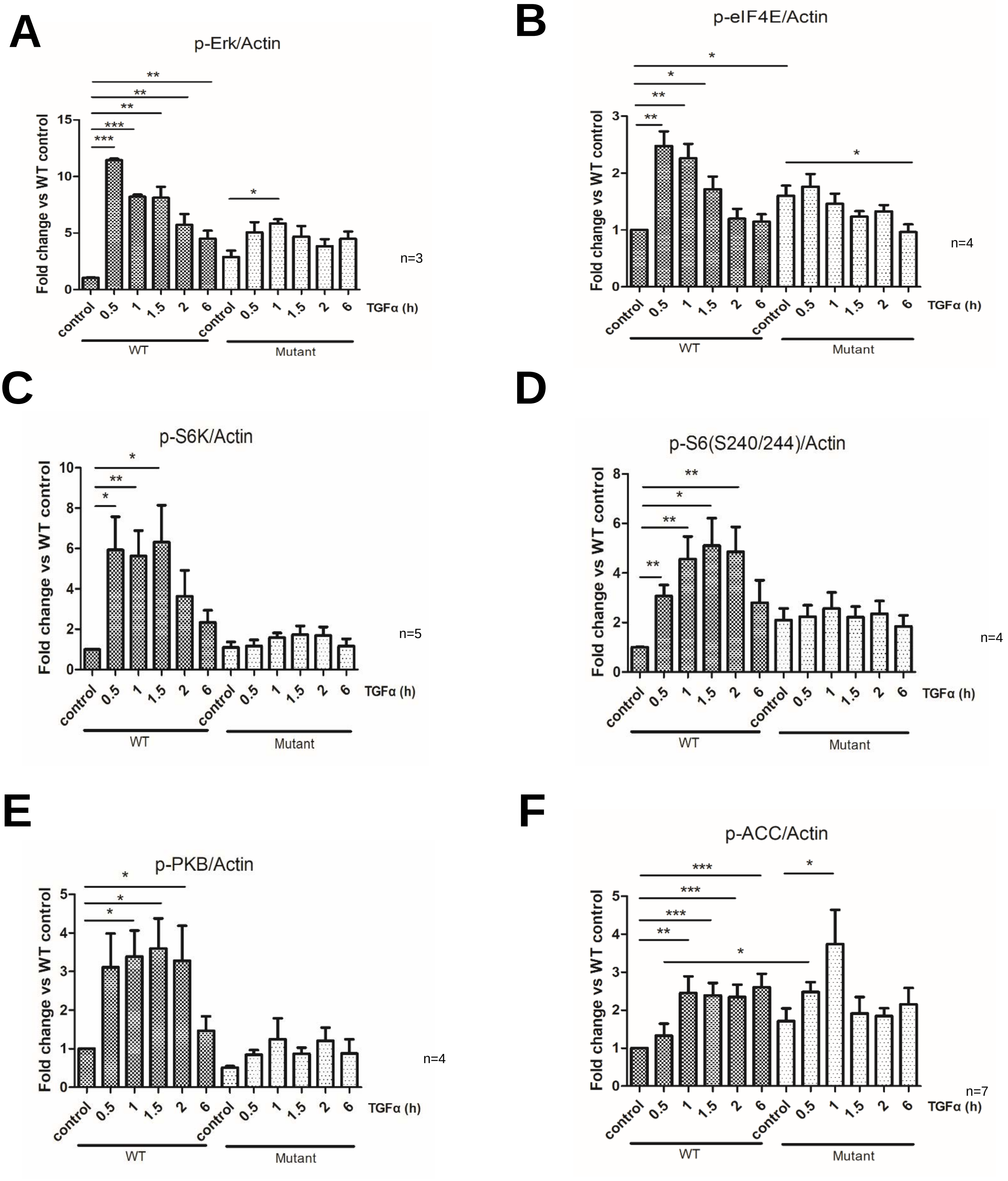

***p<0.001 ** p<0.01 * p<0.05

Supplementary Figure 8

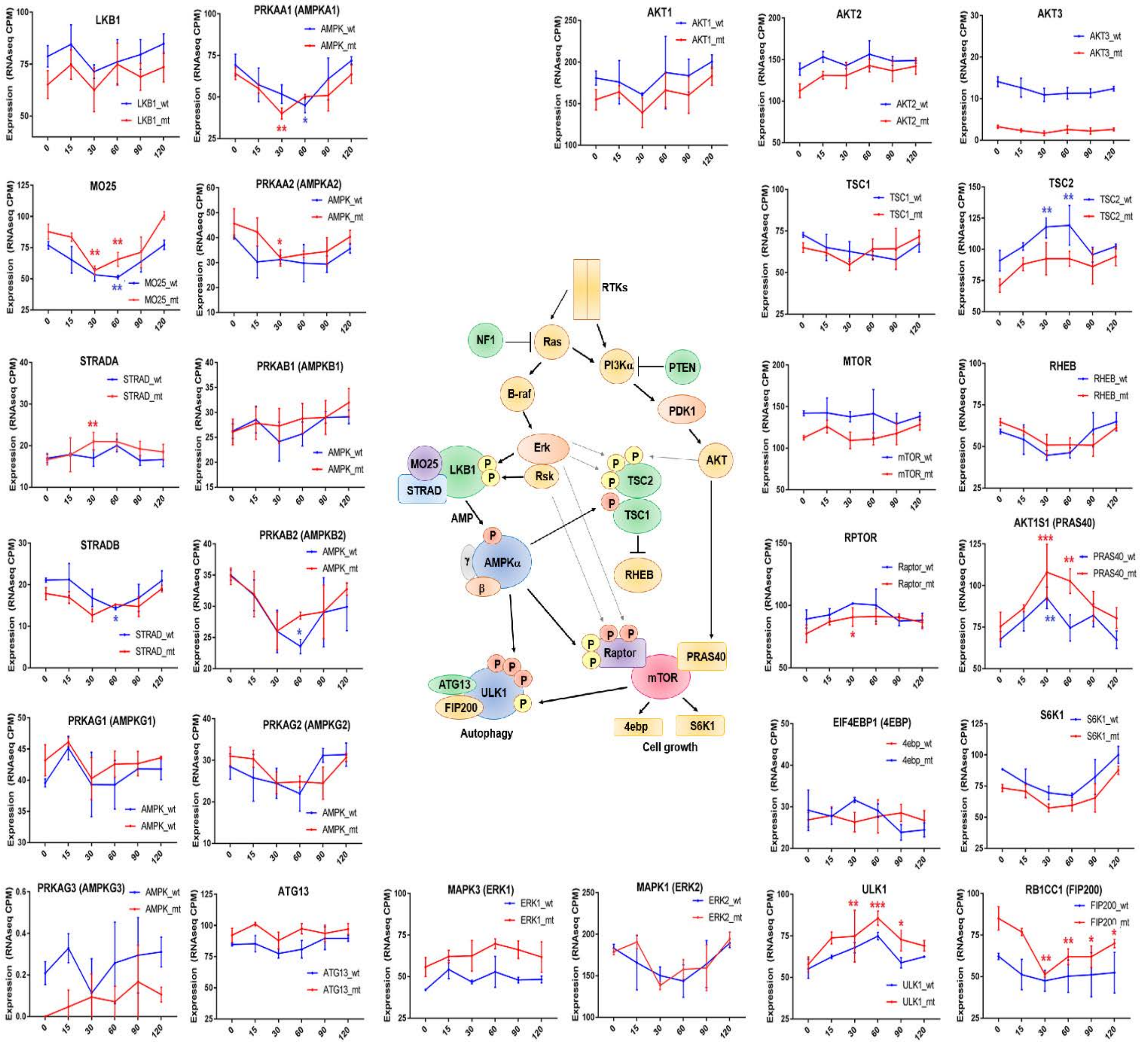

Supplementary Figure 9

A

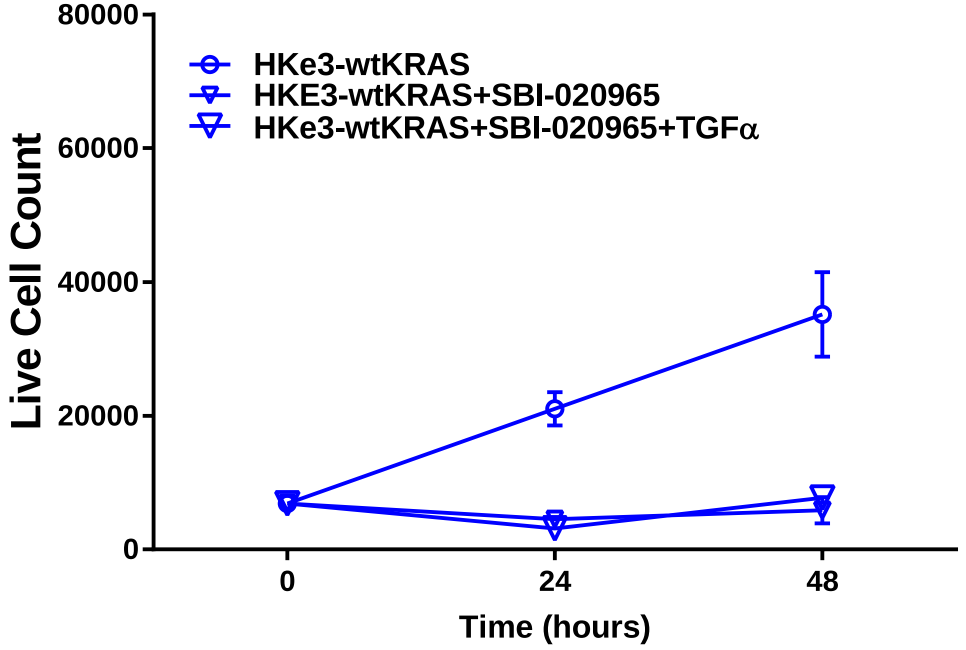

B

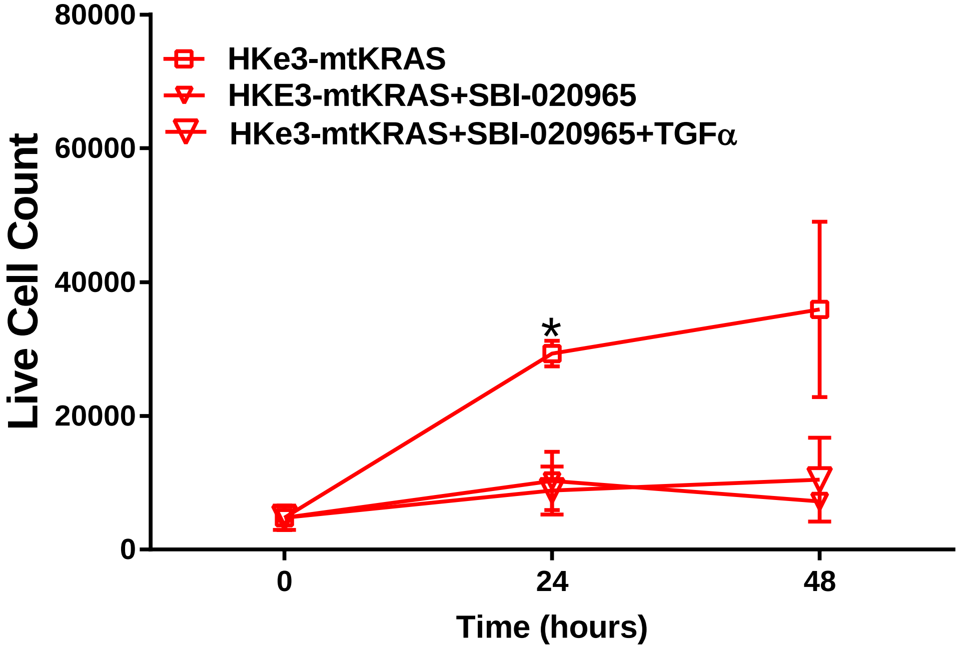

Supplement: Supplementary file 2 — Supplementary Figures [file 41416_2019_477_MOESM2_ESM.pdf]
